# UBC9 deficiency enhances immunostimulatory macrophage activation and subsequent antitumor T cell response in prostate cancer

**DOI:** 10.1172/JCI158352

**Published:** 2023-02-15

**Authors:** Jun Xiao, Fei Sun, Ya-Nan Wang, Bo Liu, Peng Zhou, Fa-Xi Wang, Hai-Feng Zhou, Yue Ge, Tian-Tian Yue, Jia-Hui Luo, Chun-Liang Yang, Shan-Jie Rong, Ze-Zhong Xiong, Sheng Ma, Qi Zhang, Yang Xun, Chun-Guang Yang, Yang Luan, Shao-Gang Wang, Cong-Yi Wang, Zhi-Hua Wang

**Affiliations:** 1 Department of Urology;; 2 Department of Thyroid and Breast Surgery;; 3 Department of Respiratory and Critical Care Medicine, Center for Biomedical Research, NHC Key Laboratory of Respiratory Diseases; and; 4 Department of Oncology; Tongji Hospital, Tongji Medical College, Huazhong University of Science and Technology, Wuhan, China.; 5 Department of Integrated Traditional Chinese and Western Medicine, Union Hospital, Tongji Medical College, Huazhong University of Science and Technology, Wuhan, China.; 6 Department of Critical Care Medicine, Zhongnan Hospital, Wuhan University, Wuhan, China.

**Keywords:** Immunology, Oncology, Cancer immunotherapy, Macrophages, Prostate cancer

## Abstract

The role of tumor-associated macrophages (TAMs), along with the regulatory mechanisms underlying distinct macrophage activation states, remains poorly understood in prostate cancer (PCa). Herein, we report that PCa growth in mice with macrophage-specific *Ubc9* deficiency is substantially suppressed compared with that in wild-type littermates, an effect partially ascribed to the augmented CD8^+^ T cell response. Biochemical and molecular analyses revealed that signal transducer and activator of transcription 4 (STAT4) is a crucial UBC9-mediated SUMOylation target, with lysine residue 350 (K350) as the major modification site. Site-directed mutation of STAT4 (K350R) enhanced its nuclear translocation and stability, thereby facilitating the proinflammatory activation of macrophages. Importantly, administration of the UBC9 inhibitor 2-D08 promoted the antitumor effect of TAMs and increased the expression of PD-1 on CD8^+^ T cells, supporting a synergistic antitumor efficacy once it combined with the immune checkpoint blockade therapy. Together, our results demonstrate that ablation of UBC9 could reverse the immunosuppressive phenotype of TAMs by promoting STAT4-mediated macrophage activation and macrophage–CD8^+^ T cell crosstalk, which provides valuable insights to halt the pathogenic process of tumorigenesis.

## Graphical abstract

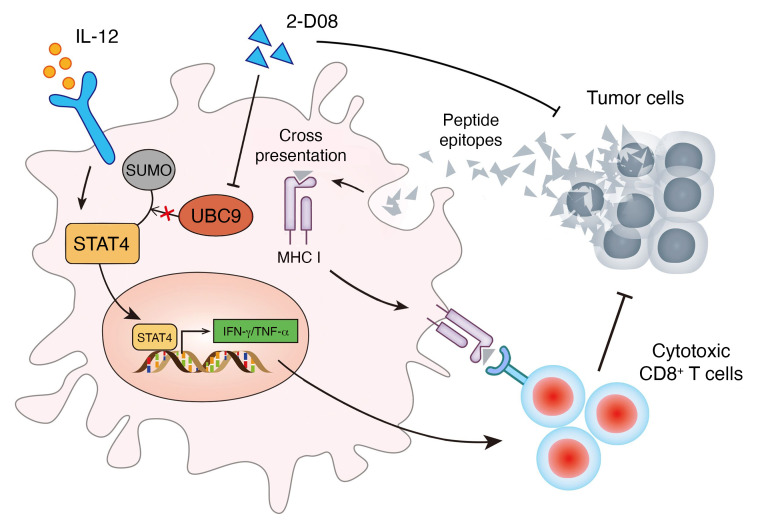

## Introduction

Prostate cancer (PCa) progression is regulated by multiple cell types existing within the tumor microenvironment (TME) (1). CD8^+^ T cells, the predominant tumoricidal effector population, are commonly present in the tumor-reactive stroma and are constantly inactivated by the surrounding coinhibitory signals (2). Immune checkpoint blockade (ICB) of PD-1/PD-L1 and CTLA-4 could reinvigorate cytotoxic CD8^+^ T cells in the TME and dampen tumor progression in numerous types of cancers (3). However, PCa is not readily responsive to ICB therapy; this is attributed to the insufficient activation of tumor-infiltrating CD8^+^ T cells (4).

There is feasible evidence that tumor-associated macrophages (TAMs) are also involved in PCa progression (5). Higher abundance of TAMs is associated with accelerated neoplastic growth, poor clinical prognosis, and treatment resistance of solid tumors (6, 7). Indeed, TAMs can directly or indirectly suppress T cell activation through insufficient antigen presentation, contact inhibition, or secretion of immune-regulatory mediators such as IL-10, IL-35, or TGF-β (7–9). Therefore, strategies aimed at targeting macrophages are considered as a promising approach to complement the existing cancer immunotherapy. However, depletion of all TAMs by disruption of the CSF1/CSF1R axis is deleterious for host defensive immunity and yields unsatisfactory results in clinical trials to date (10–12). In contrast, reprogramming TAMs from a protumoral phenotype to an antitumoral phenotype may be more feasible in clinical practice (10, 11). As a result, identification of additional factors that are able to regulate the proliferation or activation of macrophages, particularly those facilitating the phenotype for tumor antigen presentation, could help to develop new immunotherapeutics against PCa.

SUMOylation is a type of posttranslational modification that covalently attaches small ubiquitin-like modifier (SUMO) onto lysine residues of target proteins (13), thereby regulating protein stability, subcellular localization, and protein-protein interactions (14, 15). Substrates of SUMOylation encompass various signaling molecules, such as transcription factors/coregulators, RNA polymerases, histone-modifying factors, and metabolic enzymes (16, 17). SUMOylation is a multistep enzymatic process, which requires the SUMO-activating enzyme E1, the SUMO-specific conjugating enzyme E2, and the SUMO ligase E3 (18–20). UBC9, the most critical SUMO E2 conjugating enzyme, regulates macrophage function and is implicated in viral infections by supporting the survival of pathogens inside of macrophages (21–24). Previous studies, including our own, revealed that SUMOylation of KLF4 and IRF4 favors the M2 program to attenuate the development of autoimmune disorders (22, 25). Moreover, UBC9-mediated SUMOylation of IκBα prevents NF-κB activation and reduces oxidative stress and cell apoptosis (21, 23). Notably, SUMOylation also leads to an increased metastatic capacity of PCa (26, 27), and elevated expression of UBC9 is positively linked to the enhanced PCa cell proliferation (26). However, whether UBC9 regulates the polarization and functions of TAMs during PCa development remains unclear.

Herein we investigated the impact of UBC9 and its related SUMOylation process on TAMs using a PCa mouse model with macrophage-specific *Ubc9* deficiency (KO). It was found that tumor growth was markedly suppressed once SUMOylation was blocked or UBC9 was depleted in macrophages. Mechanistically, UBC9-mediated SUMOylation of signal transducer and activator of transcription 4 (STAT4) repressed macrophage activation, while inhibition of UBC9 enhanced macrophage activation and boosted the functionality of CD8^+^ T cells. Furthermore, administration of the UBC9 inhibitor 2-D08 alone reactivated the TAM–CD8^+^ T cell axis and halted PCa progression, while combination of 2-D08 with anti–PD-1 antibody further increased the overall immunotherapeutic effect. Taken together, our data prove that UBC9-mediated STAT4 SUMOylation plays a vital role in TAM-associated immunosuppression and provide an important prospect for the development of immunotherapies for PCa treatment.

## Results

### UBC9 expression is associated with defective macrophage activation and poor prognosis of PCa.

**Figure 1:**
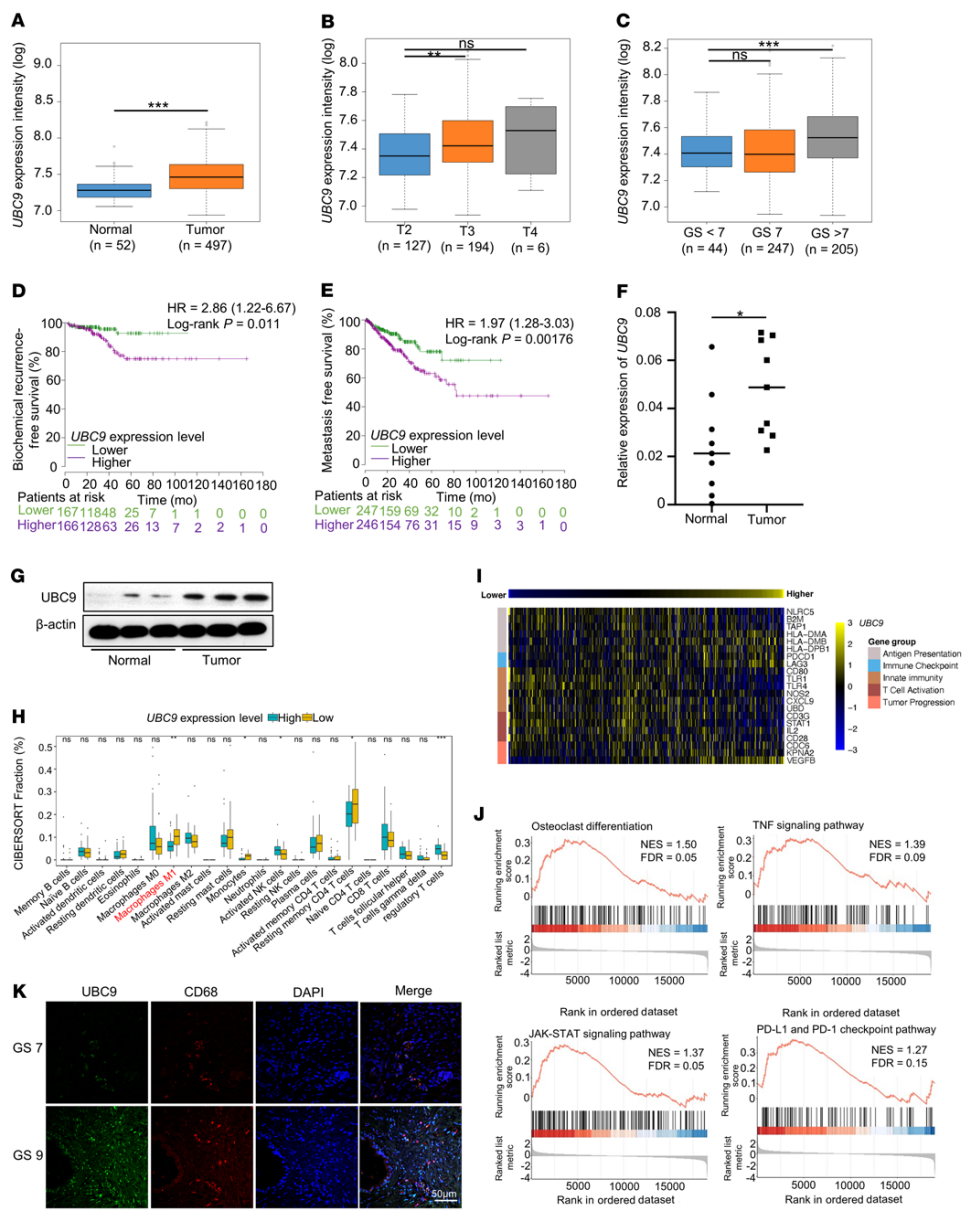
UBC9 expression is associated with defective macrophage activation and poor prognosis of PCa. (**A**) Results based on The Cancer Genome Atlas (TCGA) database showing the expression level of *UBC9* between prostate tumor and adjacent normal prostate tissue. Data are presented as median value, *n* = 549. (**B**) Results based on TCGA database indicating the expression level of *UBC9* at different stages of PCa. Data are presented as median value, *n* = 327. (**C**) Results based on TCGA database indicating the expression level of *UBC9* in different tumor grades stratified by Gleason score (GS) in PCa. Data are presented as median value, *n* = 496. (**D** and **E**) Biochemical recurrence survival rates (**D**) and metastasis-free survival rates (**E**) of PCa from TCGA database with high or low *UBC9* expression as defined by the median value. Statistical significance was determined by log-rank (Mantel-Cox) test, *n* = 497. (**F** and **G**) The expression levels of *UBC9* were identified by real-time qPCR (*n* = 9 per group) (**F**) and Western blotting (**G**). (**H**) CIBERSORT analysis characterized 22 types of immune cell composition in PCa of TCGA; *n* = 497. (**I**) Based on the TCGA database, the heatmap shows the correlation between *UBC9* expression level and genes involved in immune processes. Statistical significance was determined by Pearson’s correlation test, *n* = 497. (**J**) Gene set enrichment analysis showed the enrichment of signature genes in UBC9^hi^ prostate tumor. (**K**) Prostate tissue from PCa patients in Gleason score 7 and Gleason score 9 stained for UBC9 and CD68. Representative images for immunofluorescence staining of 5 tissue samples per group. Scale bar: 50 μm. *P < 0.05; **P < 0.01; ***P < 0.001.

To investigate the clinical relevance of SUMOylation in PCa, we focused on UBC9, the most critical SUMO E2 conjugating enzyme, and queried a series of expression and survival data sets of PCa patients. We compared UBC9 expression levels between PCa and normal tissue, and across distinct tumor stages and pathological grades. It was noted that UBC9 expression is markedly higher in PCa (Figure 1A), and is associated with advanced stages (Figure 1B) and higher Gleason score (Figure 1C). Next, we conducted survival analysis and observed that higher *UBC9* transcriptional levels were associated with poorer biochemical recurrence-free survival (Figure 1D) and metastasis-free survival (Figure 1E). In addition, real-time quantitative PCR (qPCR) and Western blotting of our in-house prostate tumor and normal tissue samples confirmed that UBC9 was upregulated in tumor tissues as compared with the adjacent normal prostate parts (Figure 1, F and G). Altogether, these results suggest that PCa patients with higher UBC9 expression display a poorer prognosis than those with lower UBC9 expression.

The above results prompted us to assess the potential contribution of UBC9 to the distinct immune subpopulations within the prostate tumor tissues. A CIBERSORT analysis was conducted and found that the classically activated macrophages (M1) were the most obviously decreased immune cell type, while regulatory T cells were the most prominently increased immune cell type, in *UBC9^hi^* PCa samples (Figure 1H). Previously, we demonstrated that UBC9-mediated SUMOylation affects the induction of regulatory T cells by modulating macrophage polarization (21); we thus assumed that TAMs play a pivotal role in the setting of innate immunity against tumor cells. We therefore next analyzed the differentially expressed genes (DEGs) among *UBC9^hi^* versus *UBC9^lo^* clusters and characterized a negative correlation for genes involved in “antigen presentation (MHC I related),” “innate immunity,” and “T cell activation”; a positive correlation with “tumor progression” and “immune checkpoint” in *UBC9^hi^* PCa subjects (Figure 1I) was also noted. Next, we performed gene set enrichment analysis and found that genes related to “osteoclast differentiation,” “TNF signaling pathway,” “JAK-STAT signaling pathway,” and “PD-1 and PD-L1 checkpoint” in cancer tissues were enriched in *UBC9^hi^* PCa subjects (Figure 1J). Finally, we carried out immunofluorescence assays by costaining UBC9 and CD68, a human macrophage marker. As expected, UBC9 was found to be highly expressed in PCa, especially in TAMs (Figure 1K). Collectively, these findings highlight an association between UBC9 expression and TAM activation relevant to its antigen-presenting capability.

### Inhibition of UBC9 halts the progression of PCa.

**Figure 2:**
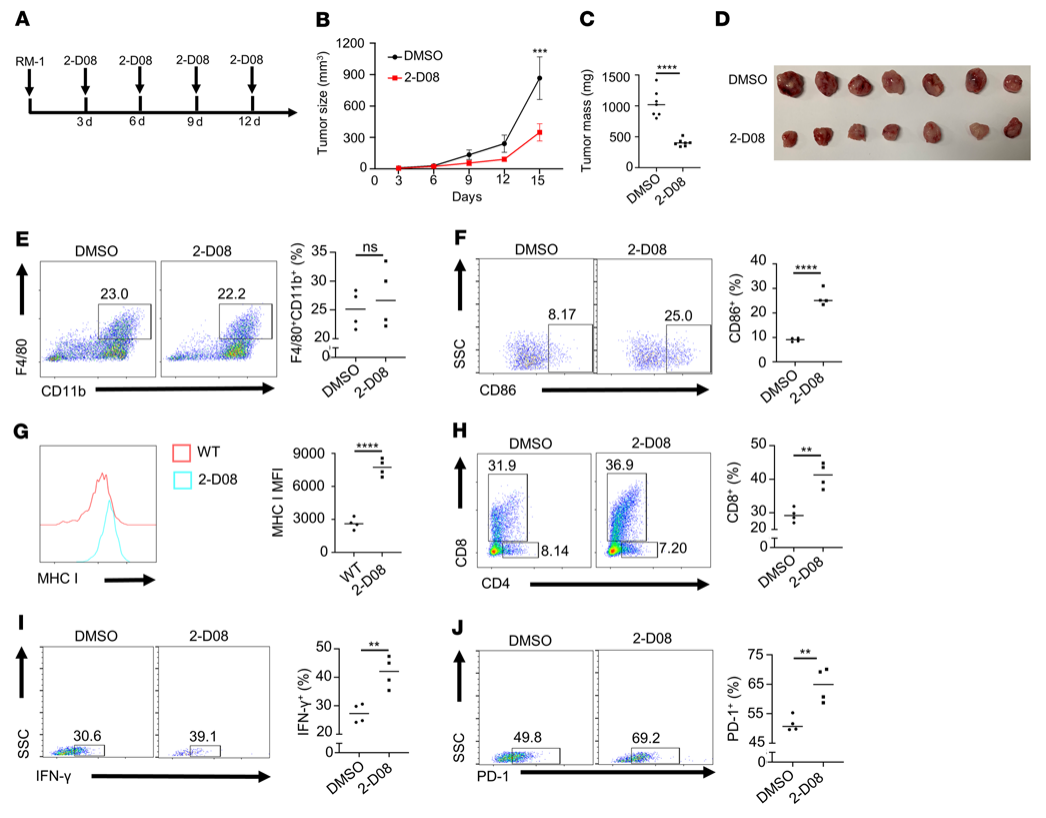
Inhibition of UBC9 represses the progression of PCa. (**A**) Schematic representation of treatment of WT mice using the UBC9 inhibitor 2-D08. (**B**) Growth of subcutaneous RM-1 tumors in WT mice treated with DMSO or 2-D08 (*n* = 7 per group). (**C** and **D**) WT mice bearing RM-1 tumors received intratumoral injections of DMSO or 2-D08. After 11 days of treatment, the mice were sacrificed, and the tumors were weighed (*n* = 7 per group). (**E**) Representative dot plots and proportions of TAMs (F4/80^+^; CD11b^+^) on day 14 (*n* = 4 per group). (**F**) Representative dot plots and proportions of CD86^+^ TAMs from tumor-bearing mice treated with DMSO or 2-D08 (*n* = 4 per group). (**G**) MHC I expression level on TAMs from tumor-bearing mice treated with DMSO or 2-D08 (*n* = 4 per group). (**H**) Representative dot plots and proportions of tumor-infiltrating CD8^+^ T cells among CD45^+^ immune cells in DMSO- or 2-D08–treated mice (*n* = 4 per group). (**I** and **J**) Representative dot plots and proportions of IFN-γ^+^ (**I**) and PD-1^+^ (**J**) tumor-infiltrating CD8^+^ T cells (*n* = 4 per group). **B** was determined by log-rank test. Data in **C** and **E**–**J** represent mean ± SEM and were analyzed by Student’s *t* test (2 tailed). **P < 0.01; ***P < 0.001; ****P < 0.0001.

To check whether UBC9 mediates PCa tumor growth in vivo, we treated RM-1 tumor–bearing C57BL/6J mice with the UBC9 inhibitor 2-D08 (10 mg/kg, every 3 days, intratumoral injection) or DMSO, the vehicle control (Figure 2A). Injection of 2-D08 markedly suppressed tumor growth (Figure 2B), reduced tumor mass (Figure 2, C and D), and increased tumor cell apoptosis as determined by TUNEL staining (Supplemental Figure 1A; supplemental material available online with this article; https://doi.org/10.1172/JCI158352DS1). Next, we analyzed the intratumoral immune cells (Supplemental Figure 1B) and found that the 2-D08–treated group possessed a higher proportion of CD45^+^ hematopoietic cells (Supplemental Figure 1C). Specifically, the frequencies of infiltrated TAMs in the 2-D08–treated and vehicle control groups were not significantly different (Figure 2E), but the frequency of CD86^+^ macrophages was higher (Figure 2F) along with elevated MHC I expression in TAMs in the 2-D08–treated group (Figure 2G). Moreover, 2-D08 treatment resulted in a higher proportion of infiltrated CD8^+^ T cells (Figure 2H). However, the frequencies of MHC II^+^ macrophages (Supplemental Figure 1D) and intratumoral CD4^+^ T cells (Supplemental Figure 1E) showed no difference between the 2 groups, but the frequency of activated CD8^+^ T cells was increased in the 2-D08–treated group (Figure 2I and Supplemental Figure 1F). Intriguingly, we also noted that PD-1^+^ CD8^+^ T cells were markedly increased in the 2-D08–treated group (Figure 2J), which may be secondary to the heightened T cell activation status.

Given that prostate tumor cells also express UBC9, we next sought to check the possible direct impact of 2-D08 on prostate tumor growth. To this end, we first examined tumor cell growth upon treatment with 2-D08 and TAK-981, a selective and potent small-molecule inhibitor of the SUMO-activating enzyme E1, respectively. The growth of prostate tumor cells was impeded by both 2-D08 (targeting E2) and TAK-981 (targeting E1) in a dose-dependent manner (Supplemental Figure 2, A and B). Consistently, the development of subcutaneously inoculated prostate tumors (Supplemental Figure 2, C–E) and B16 melanomas (Supplemental Figure 2, F–H) in T cell–deficient nude mice was restrained by TAK-981 treatment. To further test the antitumor effect and specificity of 2-D08, we inoculated nude mice with PCa cells transduced by *Ubc9*-overexpressing adenovirus or empty vector (Supplemental Figure 2I) before the administration of 2-D08 or control vehicle. Ectopic *Ubc9* expression promoted tumor growth, while injection of 2-D08 markedly attenuated tumor growth, in nude mice (Supplemental Figure 2J). Particularly, the *Ubc9-*overexpressing tumors treated with 2-D08 exhibited growth comparable to that seen in the control group, indicating that 2-D08 is a specific UBC9 inhibitor (Supplemental Figure 2, K and L). Taken together, these data support that the UBC9 inhibitor impedes PCa progression not only by promoting TAM and CD8^+^ T cell activation, but also by directly suppressing the growth of tumor cells.

### Ubc9 deficiency in macrophages suppresses the progression of PCa.

**Figure 3:**
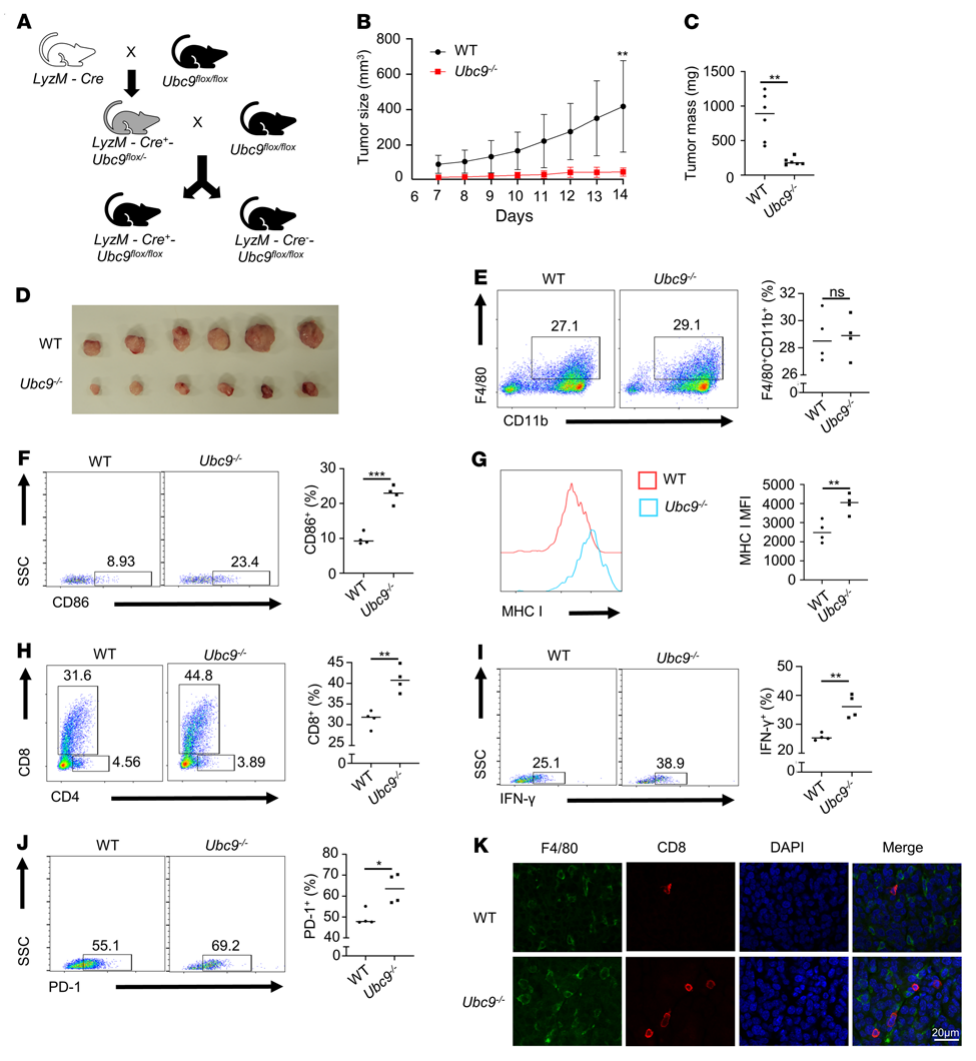
*Ubc9* deficiency in macrophages suppresses the progression of PCa. (**A**) *Ubc9^fl/fl^* mice were crossed with *LyzM-Cre* transgenic mice to generate the macrophage-specific *Ubc9*-knockout mice, which were denoted as *LyzM-Cre^+^Ubc9^fl/fl^*. (**B**–**D**) Tumor growth curve (**B**) and tumor mass (**C** and **D**) at day 14 in WT and *Ubc9^–/–^* groups (*n* = 7 per group). (**E**) Proportion of TAMs among CD45^+^ immune cells from WT and *Ubc9^–/–^* groups (*n* = 4 per group). (**F**) Proportion of CD86^+^ TAMs in WT and *Ubc9^–/–^* groups (*n* = 4 per group). (**G**) MHC I expression level on TAMs in WT and *Ubc9^–/–^* groups (*n* = 4 per group). (**H**) Proportion of tumor-infiltrating CD8^+^ T cells among CD45^+^ immune cells in WT and *Ubc9^–/–^* groups (*n* = 4 per group). (**I** and **J**) Proportion of IFN-γ^+^ (**I**) and PD-1^+^ (**J**) tumor-infiltrating CD8^+^ T cells in WT and *Ubc9^–/–^* groups (*n* = 4 per group). (**K**) Tumors from WT and *Ubc9^–/–^* mice were isolated, fixed, embedded in paraffin, sectioned, and stained for F4/80 (green) and CD8 (red). Similar results were obtained from 3 independent experiments. **B** was determined by log-rank test. Data in **C** and **E**–**J** represent mean ± SEM and were analyzed by Student’s *t* test. Scale bar: 20 μm. *P < 0.05; **P < 0.01; ***P < 0.001.

To further determine the role of TAMs in UBC9-mediated PCa progression, we constructed a *LyzM-Cre-Ubc9-loxP* macrophage conditional knockout mouse model bearing prostate tumor (Figure 3A and Supplemental Figure 3A). It was found that the implanted prostate tumors grew much slower (Figure 3B) and were smaller (Figure 3, C and D) in *Ubc9^–/–^* mice. Moreover, TUNEL staining revealed that the *Ubc9^–/–^* group was characterized by increased apoptosis (Supplemental Figure 3B) coupled with higher infiltration of immune cells (Supplemental Figure 3C). However, no difference was detected in terms of infiltrated TAMs between the 2 groups (Figure 3E). In contrast, the proportion of CD86^+^ TAMs (Figure 3F) and the expression level of MHC I in TAMs (Figure 3G) were substantially higher in *Ubc9^–/–^* mice, while the percentages of MHC II^+^ TAMs were similar between the 2 groups of mice (Supplemental Figure 3D). Importantly, the *Ubc9^–/–^* mice manifested a similar proportion of tumor-infiltrating CD4^+^ T cells (Supplemental Figure 3E) but a markedly higher proportion of intratumoral CD8^+^ T cells (Figure 3H and Supplemental Figure 3F). In line with those observations, the percentage of IFN-γ^+^ CD8^+^ T cells was enhanced in the tumors isolated from *Ubc9^–/–^* mice (Figure 3I), and a higher proportion of PD-1–expressing CD8^+^ T cells in tumors from *Ubc9*^–/–^ mice was also observed (Figure 3J). By immunofluorescence staining, we found that TAMs were in close proximity with tumor-infiltrating CD8^+^ T cells (Figure 3K), implying their intimate interaction. Collectively, these results suggest that *Ubc9* deficiency in macrophages remarkably attenuates PCa progression by mobilizing antitumor CD8^+^ T cells.

### Loss of Ubc9 enhances macrophage M1 program.

**Figure 4:**
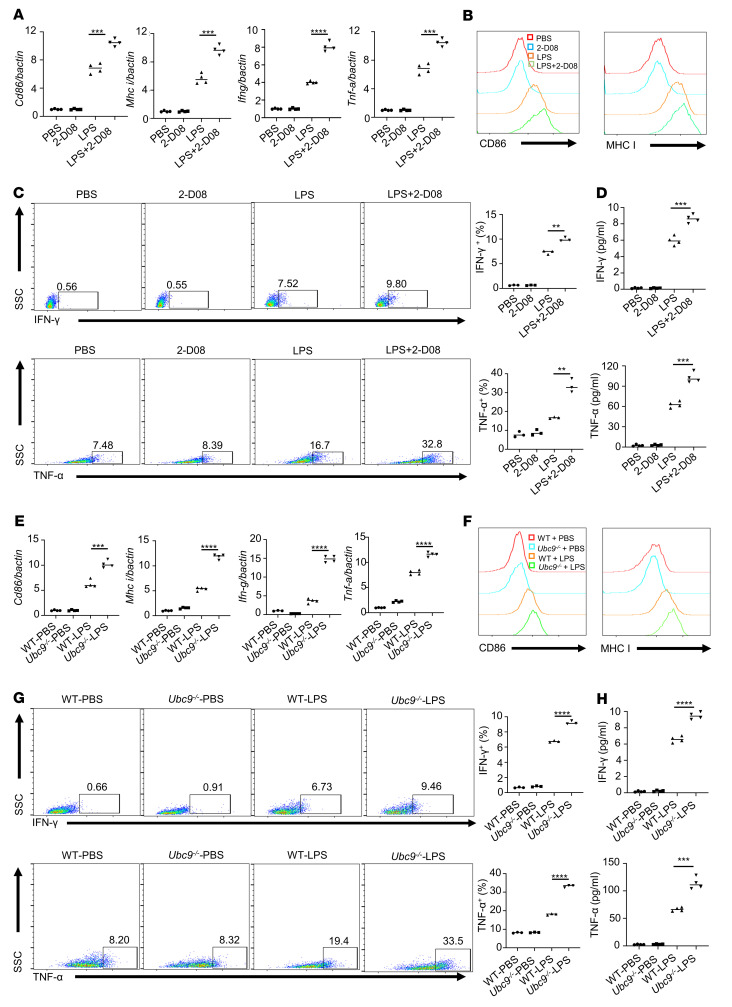
Loss of *Ubc9* facilitates macrophage activation. (**A**) Real-time qPCR analysis of *Cd86*, *Mhc-i*, *Ifn-g*, and *Tnf-a* in macrophages treated with PBS, 2-D08, LPS, and LPS plus 2-D08. (**B**) Representative histogram measuring the expression levels of CD86 and MHC I and the MFI of each marker in the above-mentioned 4 groups. (**C**) Proportions of IFN-γ^+^ and TNF-α^+^ macrophages in the above-mentioned 4 groups. (**D**) ELISA analysis of secreted IFN-γ and TNF-α in the 4 groups of macrophages. (**E**) Real-time qPCR analysis of *Cd86*, *Mhc-i*, *Ifn-g*, and *Tnf-a* in WT and *Ubc9^–/–^* macrophages treated with PBS or LPS. (**F**) Representative histogram measuring the expression levels of CD86 and MHC I and the MFI of each marker in WT and *Ubc9^–/–^* macrophages treated by PBS or LPS. (**G**) Proportions of IFN-γ^+^ and TNF-α^+^ cells in WT and *Ubc9^–/–^* macrophages treated with PBS or LPS. (**H**) ELISA analysis of secreted IFN-γ and TNF-α in the 2 groups of macrophages treated with PBS or LPS. Data represent mean ± SEM and were analyzed by Student’s *t* test (*n* = 4 per comparison group). Similar results were obtained from 3 independent experiments. **P < 0.01; ***P < 0.001; ****P < 0.0001.

Classic activation of (M1) macrophages leads to the upregulation of proinflammatory cytokines and surface costimulatory molecules, which are essential for the optimal antitumor response (28). It was noted that pretreatment of LPS-stimulated bone marrow–derived macrophages (BMDMs) with 2-D08 induced much higher expression of CD86, MHC I, and proinflammatory cytokines such as IFN-γ and TNF-α as determined by real-time qPCR assays (Figure 4A). Consistently, flow cytometry analysis of CD86, MHC I (Figure 4B), IFN-γ, and TNF-α (Figure 4C) revealed their relative abundance in 2-D08–treated BMDMs, and a higher concentration of IFN-γ and TNF-α was also detected in the corresponding culture supernatant by ELISA measurement (Figure 4D). To further substantiate the above findings, we compared the activation markers of WT and *Ubc9*-deficient BMDMs differentiated under M1 condition. Similarly, markedly enhanced expression of CD86, MHC I, and proinflammatory cytokines (IFN-γ and TNF-α) was observed in the *Ubc9^–/–^* BMDMs as determined by real-time qPCR (Figure 4E), flow cytometry (Figure 4, F and G), and ELISA analyses (Figure 4H). Together, our data support that *Ubc9* deficiency promotes macrophage M1 program related to antitumor response.

### Macrophages deficient in Ubc9 are potent to activate antigen-specific CD8^+^ T cells.

**Figure 5:**
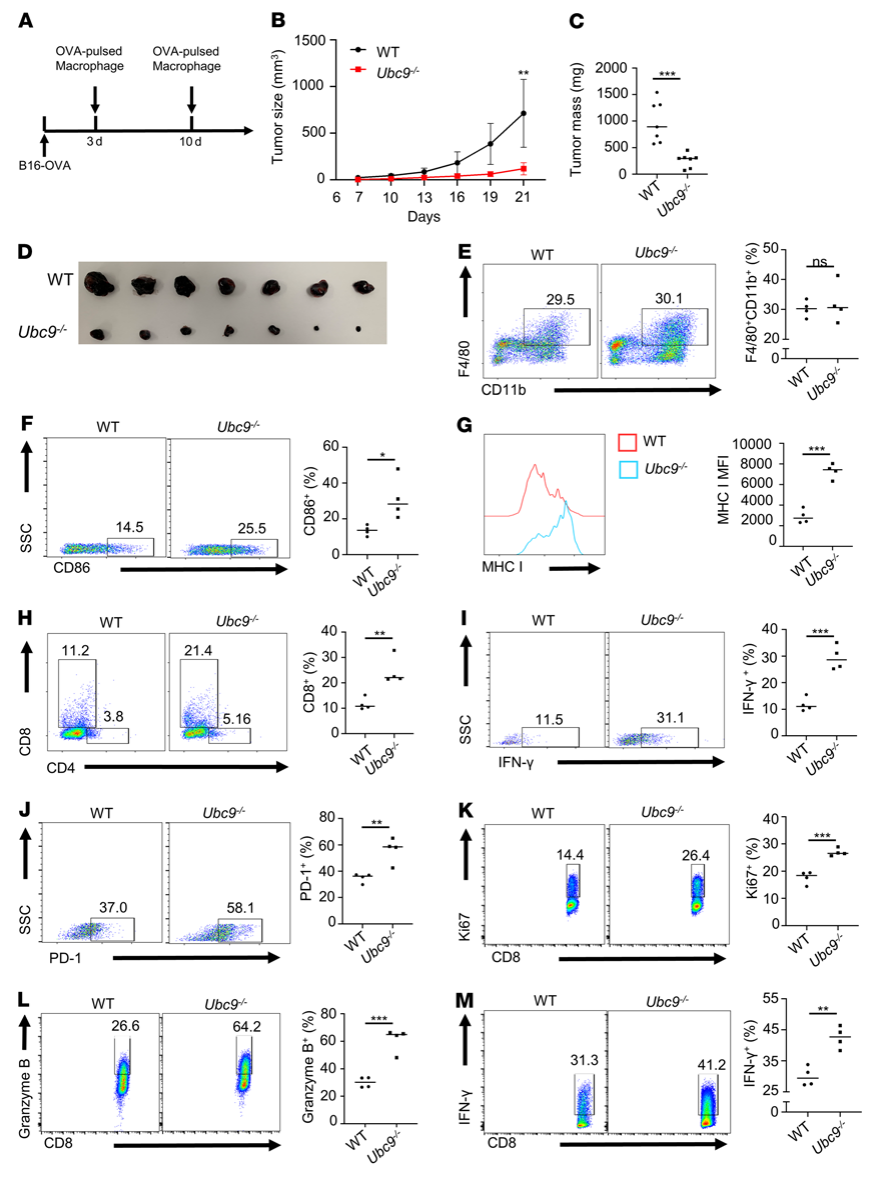
Loss of *Ubc9* in macrophages activates antigen-specific CD8^+^ T cells. (**A**) Experimental design for macrophage adoptive transfer in mice bearing B16-OVA tumors. (**B**–**D**) Tumor growth curve (**B**) and tumor mass (**C** and **D**) of B16-OVA melanoma at day 14 in the 2 groups transferred with OVA-pulsed WT or *Ubc9^–/–^* macrophages (*n* = 7 per group). (**E**) Proportion of TAMs among CD45^+^ immune cells in these 2 groups (*n* = 4 per group). (**F**) Proportion of CD86^+^ TAMs in these 2 groups (*n* = 4 per group). (**G**) MHC I expression level on TAMs in these 2 groups (*n* = 4 per group). (**H**) Proportion of tumor-infiltrating CD8^+^ T cells among CD45^+^ immune cells in these 2 groups (*n* = 4 per group). (**I** and **J**) Proportions of IFN-γ^+^ (**I**) and PD-1^+^ (**J**) tumor-infiltrating CD8^+^ T cells in these 2 groups (*n* = 4 per group). (**K**–**M**) Proportions of Ki67^+^ (**K**), granzyme B^+^ (**L**), and IFN-γ^+^ (**M**) CD8^+^ T cells in coculture assay with OVA-pulsed WT or *Ubc9^–/–^* macrophages and OT-I CD8^+^ T cells (*n* = 4 per group). **B** was determined by log-rank test. Data in **C** and **E**–**M** represent mean ± SEM and were analyzed by Student’s *t* test. *P < 0.05; **P < 0.01; ***P < 0.001.

To determine the impact of *Ubc9* on the functionality of macrophages to activate effector CD8^+^ T cells, B16-OVA melanoma–bearing mice were employed. Briefly, WT and *Ubc9^–/–^* macrophages pulsed by LPS along with OVA were adoptively transferred adjacent to the B16-OVA tumor intradermally on days 3 and 10 (Figure 5A). Similar to the results described above, the tumors transferred with *Ubc9^–/–^* macrophages were characterized by attenuated growth rate (Figure 5B) and smaller size (Figure 5, C and D), coupled with repressed tumor cell proliferation (Supplemental Figure 4A) and increased apoptosis (Supplemental Figure 4B) along with enhanced CD45^+^ immune cell infiltration (Supplemental Figure 4C). Interestingly, no perceptible difference in the proportion of TAMs was detected between the 2 groups of mice (Figure 5E). However, a higher proportion of CD86^+^ TAMs (Figure 5F) and higher MHC I expression (Figure 5G) were observed in *Ubc9^–/–^* macrophages, but no difference in MHC II expression was noted in comparison with the WT counterparts (Supplemental Figure 4D). The proportion of intratumoral CD4^+^ T cells was comparable (Supplemental Figure 4E), but a substantially higher proportion of intratumoral CD8^+^ T cells was detected in the *Ubc9^–/–^* group (Figure 5H and Supplemental Figure 4F), which was characterized by enhanced IFN-γ (Figure 5I) and PD-1 (Figure 5J) expression. These data indicate that *Ubc9^–/–^* macrophages are more potent in activating tumor-infiltrating CD8^+^ T cells.

To further confirm the above observations, we cocultured WT or *Ubc9*-deficient OVA-pulsed macrophages with OT-I CD8^+^ T cells. As expected, the *Ubc9^–/–^* macrophages dramatically enhanced Ki67 (Figure 5K), granzyme B (Figure 5L), and IFN-γ (Figure 5M) expression in CD8^+^ T cells compared with the WT controls. To exclude the possibility that tumor cells directly restrain CD8^+^ T cell–mediated immunosurveillance, we treated B16-OVA cells in the presence or absence of the UBC9 inhibitor 2-D08, followed by coculturing with OT-I CD8^+^ T cells. It was noted that the activation of CD8^+^ T cells was comparable and relatively low in both groups of cells (Supplemental Figure 4, G–I), indicating that UBC9-associated CD8^+^ T cell repression within the tumors was predominantly shaped by TAMs rather than by tumor cells. Collectively, our data demonstrate that loss of *Ubc9* promotes macrophage M1 program along with enhanced antigen presentation capacity, thereby inducing CD8^+^ T cell activation.

### UBC9-mediated STAT4 SUMOylation inhibits macrophage M1 program.

**Figure 6:**
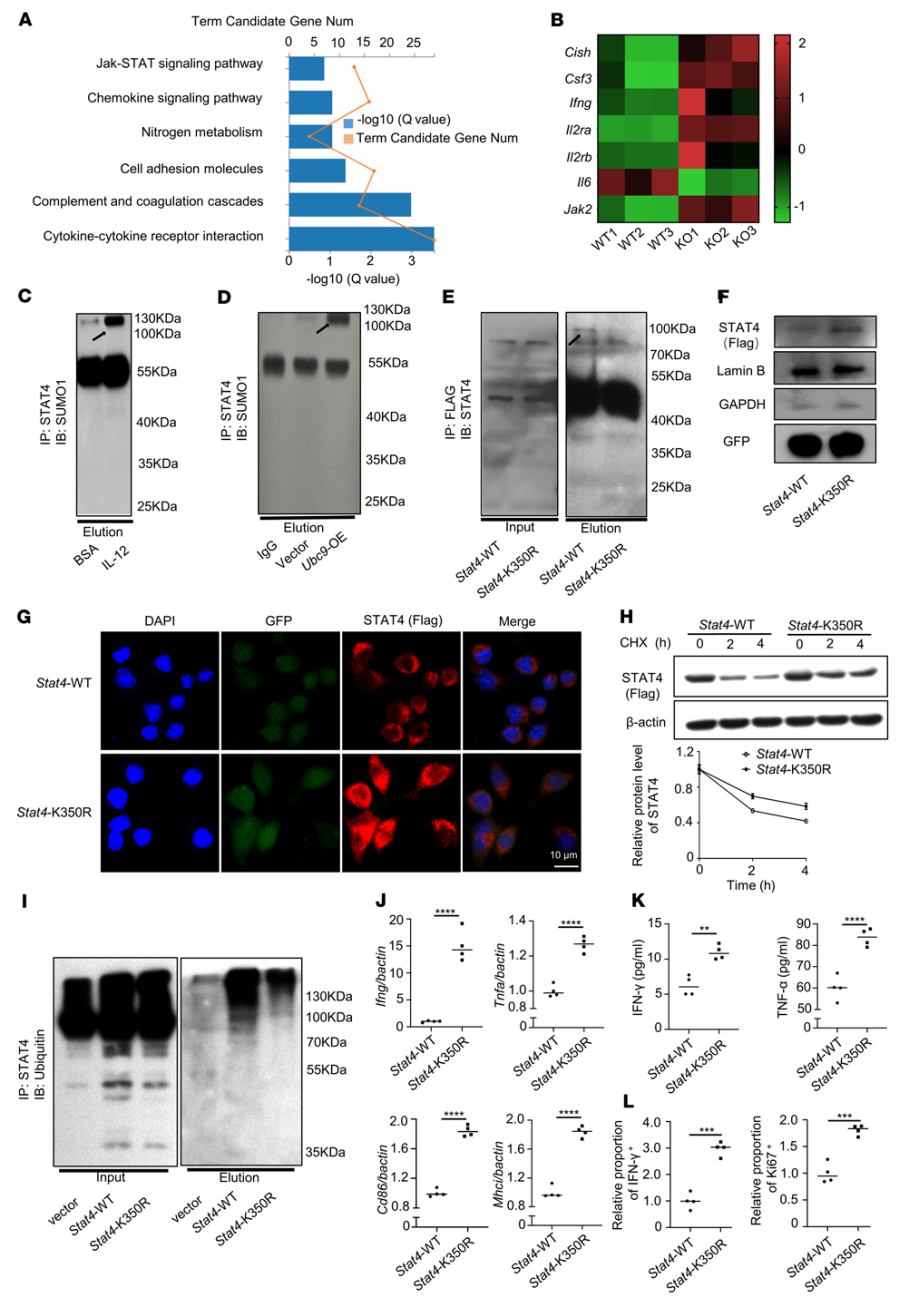
UBC9-mediated STAT4 SUMOylation inhibits macrophage activation. (**A**) TAMs were sorted and analyzed by RNA-Seq from WT and *Ubc9^–/–^* PCa-bearing mice. Gene Ontology analysis demonstrated the enriched pathways in TAMs. (**B**) Heatmap showing genes associated with the JAK/STAT signaling pathway. (**C**) Immunoprecipitation identified STAT4 SUMOylation in BMDMs treated with BSA or IL-12. (**D**) SUMOylated STAT4 was obviously detected in BMDMs transduced with *Ubc9*-overexpressing (*Ubc9*-OE) adenovirus. (**E**) SUMOylated STAT4 was detected in BMDMs transduced with FLAG-tagged *Stat4*-WT but not *Stat4*-K350R after endogenous *Stat4* was knocked down. (**F**) Western blotting was used to analyze nuclear STAT4 expression in macrophages transduced with FLAG-tagged *Stat4*-WT and *Stat4*-K350R adenoviruses after endogenous *Stat4* was knocked down by siRNA. (**G**) Macrophages transduced with FLAG-tagged *Stat4*-WT and *Stat4*-K350R after knockdown of endogenous *Stat4* were costained with anti-FLAG (red) and DAPI (blue) and imaged with confocal microscopy. (**H**) Transduced macrophages were pretreated with CHX for the indicated times, and STAT4 (FLAG-tagged) protein levels were analyzed. (**I**) Transduced macrophages were pretreated with CHX plus MG-132, and immunoprecipitation was conducted to identify ubiquitination level of STAT4 in the 2 groups. (**J**) Transcription levels of *Ifn-g*, *Tnf-a*, *Cd86*, and *Mhc-i* in virus-transduced macrophages quantified by real-time qPCR (*n* = 4 per group). (**K**) ELISA was conducted to check the secreted IFN-γ and TNF-α of virus-transduced macrophages (*n* = 4 per group). (**L**) Macrophages transduced with *Stat4*-WT or *Stat4*-K350R after endogenous *Stat4* knockdown were cocultured with CD8^+^ T cells in the presence of anti-CD3. The proportions of Ki67^+^ and IFN-γ^+^ CD8^+^ T cells were examined (*n* = 4 per group). **C**–**I** represent at least 2 independent experiments. Data in **J**–**L** represent mean ± SEM and were analyzed by Student’s *t* test. **P < 0.01; ***P < 0.001; ****P < 0.0001.

To further explore the mechanisms by which UBC9 inhibits macrophage M1 program, TAMs from the WT and *Ubc9^–/–^* prostate tumor–bearing mice were sorted for transcriptomic analysis. Gene Ontology (GO) analysis identified the JAK/STAT signaling pathway as the most prominent one enriched by the DEGs upregulated in the *Ubc9^–/–^* TAMs (Figure 6, A and B). Since STAT4 is a critical transcription factor to mediate IL-12–dependent IFN-γ production in macrophages (29), we thus checked whether UBC9 mediates STAT4 SUMOylation following IL-12 stimulation. BMDMs were stimulated with BSA or IL-12, and then subjected to immunoprecipitation using a STAT4 antibody, and the resulting products were probed with a SUMO1 antibody. A reactive band with molecular weight of STAT4 plus SUMO1 was more obviously detected in IL-12–stimulated BMDMs (Figure 6C, arrow). Moreover, the SUMOylation of STAT4 was further confirmed in BMDMs with ectopic UBC9 expression (Figure 6D). Bioinformatics analysis using SUMOsp 2.0 software revealed that lysine 350 (K350) could be the major SUMOylation site (Supplemental Figure 5A). Next, adenoviruses carrying FLAG-tagged *Stat4*-WT and *Stat4*-K350R (the lysine residue at position 350 was replaced by arginine) were transduced into BMDMs, followed by IL-12 stimulation, and then subjected to immunoprecipitation using a FLAG antibody. As expected, the SUMO1 reactive band disappeared once lysine 350 was mutated (Figure 6E).

To check the functional impact of SUMOylation on STAT4, the transduced BMDMs described above, after IL-12 stimulation, were collected for analysis of ectopic STAT4 subcellular location using the FLAG antibody. A markedly higher nuclear proportion of ectopic STAT4 was detected in *Stat4*-K350R–transduced cells as determined by immunoblotting and immunofluorescence staining (Figure 6, F and G), indicating that SUMOylation represses STAT4 nuclear translocation. To further check whether SUMOylation affects STAT4 stability, the above transduced BMDMs were treated with cycloheximide to block protein synthesis, followed by analysis of ectopic STAT4 levels. Much higher levels of ectopic STAT4 were detected in *Stat4*-K350R–transduced cells (Figure 6H), which prompted us to check its ubiquitination status. To this end, the cells were treated with MG-132, a proteasome inhibitor. Indeed, SUMOylation markedly enhanced the ubiquitination level of STAT4 (Figure 6I), which facilitates the proteasome-dependent degradation machinery. In line with these observations, the *Stat4*-K350R–transduced cells displayed higher IFN-γ and TNF-α expression at both the mRNA and the protein level (Figure 6, J and K).

To determine the functional impact of STAT4 SUMOylation on macrophages against tumor development, BMDMs were first transfected with a *Stat4* siRNA within the 3′-untranslated region to knock down the endogenous *Stat4* (Supplemental Figure 5B), and then transduced with *Stat4*-WT or *Stat4*-K350R adenoviruses, followed by coculturing with CD8^+^ T cells. Markedly higher CD8^+^ T cell proliferation and activation were noted in the cultures with *Stat4*-K350R–transduced BMDMs (Figure 6L and Supplemental Figure 5, C and D). To confirm this observation, we adoptively transferred *Stat4*-K350R– or *Stat4*-WT–transduced macrophages into the adjacent sites of prostate tumors. Similarly to the results described above, the growth of prostate tumors was dramatically repressed in mice with transferred *Stat4*-K350R–transduced BMDMs compared with the WT counterparts (Supplemental Figure 5, E–G). Collectively, these findings support that SUMOylation of STAT4 at lysine 350 suppresses the stability and transcriptional activity of STAT4.

### Inhibition of UBC9 represses prostate tumor growth synergistically with anti–PD-1 therapy.

**Figure 7:**
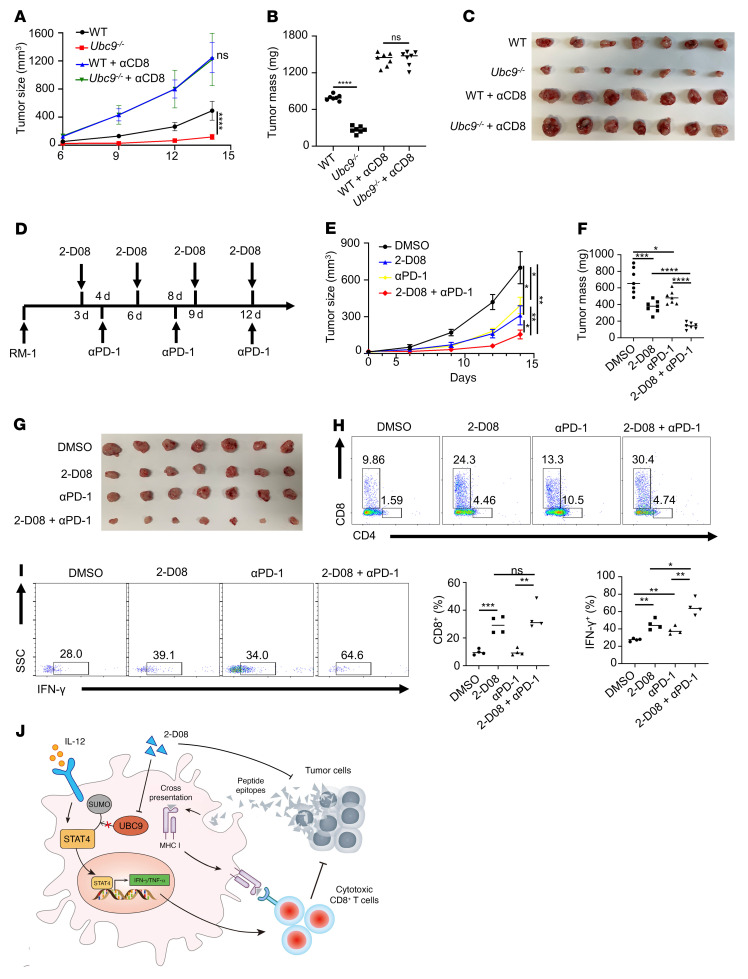
Inhibition of UBC9 represses prostate tumor growth synergistically with anti–PD-1 therapy. (**A**–**C**) Tumor growth curve (**A**) and tumor mass (**B** and **C**) following CD8^+^ T cell depletion at day 14 in WT and *Ubc9^–/–^* mice bearing PCa tumors (*n* = 7 per group). (**D**) Experimental design of combined therapy using 2-D08 and anti–PD-1 antibody in prostate tumors. (**E**–**G**) Prostate tumor growth curve (**E**) and tumor mass (**F** and **G**) at day 14 in PCa-bearing mice treated with DMSO, 2-D08, anti–PD-1 antibody, or 2-D08 plus anti–PD-1 antibody (*n* = 7 per group). (**H**) Percentage of CD4^+^ and CD8^+^ T cells among CD45^+^ immune cells in the 4 groups (*n* = 4 per group). (**I**) Proportions of IFN-γ^+^ CD8^+^ T cells in the 4 groups (*n* = 4 per group). **A** and **E** were determined by log-rank test. Data in **B**, **F**, and **I** represent mean ± SEM and were analyzed by Student’s *t* test. *P < 0.05; **P < 0.01; ***P < 0.001; ****P < 0.0001. (**J**) Schematic model depicting the antitumor effect of UBC9 inhibition in TAMs. Inhibition of STAT4 SUMOylation leads to TAM activation and enhanced antigen cross-presentation to CD8^+^ T cells, which are responsible for the cytotoxicity on PCa cells. Alternatively, the UBC9 inhibitor 2-D08 exerts a direct tumor-killing effect, leading to the release of tumor-associated antigens.

Finally, we sought to translate the above findings into a clinical setting. We first checked the impact of CD8^+^ T cell depletion on tumor growth. A CD8^+^ T cell–deficient PCa model was generated, and CD8^+^ T cells were depleted using a CD8a neutralizing antibody (Supplemental Figure 6A). Unsurprisingly, deletion of CD8^+^ T cells led to the fastest tumor growth and almost abolished the therapeutic effect of macrophage-specific *Ubc9* deficiency (Figure 7, A–C), which further supported the notion that the antitumor effect of TAMs is predominantly due to the priming of CD8^+^ T cells.

Given that *Ubc9^–/–^* TAMs upregulated PD-1 expression on CD8^+^ T cells, we assumed that UBC9 inhibitor represses PCa synergistically with anti–PD-1 therapy. To this end, the therapeutic efficacies of combinatorial therapy (2-D08 plus anti–PD-1) versus monotherapies (2-D08 or anti–PD-1 alone) in a PCa model were assessed (Figure 7D). Remarkably, 2-D08 plus anti–PD-1 suppressed prostate tumor progression with much higher efficacy than either 2-D08 or anti–PD-1 alone (Figure 7, E–G). Moreover, the combinatorial therapy was more potent to attenuate prostate tumor proliferation and to enhance tumor cell apoptosis than the monotherapies (Supplemental Figure 6, B and C). A more pronounced increase of CD8^+^ T cell frequency (Figure 7H and Supplemental Figure 6D) along with a higher percentage of IFN-γ^+^ T cells among CD8^+^ T cells (Figure 7I) was also observed in the prostate tumors following 2-D08 plus anti–PD-1 treatment. Collectively, these findings demonstrate that the UBC9 inhibitor combined with a PD-1 blocking antibody could be a viable strategy against PCa in clinical settings.

## Discussion

In this report, we demonstrate convincing evidence that elevated UBC9 expression is linked to defective activation of TAMs and restrained intratumoral CD8^+^ T cell response in PCa. UBC9 mediates the SUMOylation of STAT4 at lysine residue 350, by which it facilitates ubiquitination-dependent degradation of STAT4 and inhibits STAT4-mediated macrophage M1 polarization. More importantly, the UBC9 inhibitor 2-D08 combined with the PD-1 blocking antibody substantially enhanced CD8^+^ T cell cytotoxicity and improved the effectiveness of PCa therapy (Figure 7J).

Myeloid cells, T cells, and B cells are found in the PCa TME, while these immune cells lack sufficient antitumor potency (30, 31). Macrophages are a plastic cell population manifested by a spectrum of polarization features or activation states in TME (32). In progressing tumors, the dominant TAM phenotype is antiinflammatory or immune-regulatory instead of proinflammatory and tumoricidal. A series of TAM-targeting strategies have been investigated in preclinical models, including macrophage depletion, inhibition of macrophage recruitment, and macrophage reprogramming (33). Recently, a growing body of evidence has emphasized the epigenetic regulation of macrophage function, including long-distance chromatin remodeling, DNA methylation, posttranslational modifications, and noncoding RNAs (34). Our study uncovered that the UBC9 inhibitor 2-D08 possesses high potency to suppress PCa cell proliferation and to promote TAM function, but has no effect on tumor cell–mediated CD8^+^ T cell activation. To our knowledge, this is the first report revealing that UBC9 regulates PCa progression via TAMs, which paves a new therapeutic avenue to curb cancer development.

The JAK/STAT signaling pathway plays a crucial role in immune cell activation and is tightly regulated by SUMOylation machinery (35). PIAS1 mediates SUMOylation of STAT1, which selectively regulates a subset of IFN-inducible genes by disturbing the recruitment of STAT1 to the promoter regions in macrophages (36, 37). PIAS3 binds to activated STAT3 to interfere with its DNA-binding ability (38), and SUMOylation of STAT5 causes the transcriptional repression of STAT5-driven genes (39, 40). Notably, STAT4 inhibits macrophage polarizing to an M2-like protumorigenic phenotype (41), thereby facilitating macrophage proinflammatory function (42). However, whether SUMOylation is involved in the regulation of STAT4 activity, and its relevance in macrophage activation, particularly in a PCa setting, have yet to be addressed.

There is compelling evidence that SUMO modifications occur upon macrophage activation, which serve as a mechanism to modulate innate immune response (20, 43, 44). SUMOylation of AKT, KLF4, PKM2, PPARγ, and TEFB modulates macrophage polarization (45), and the deSUMOylation of MKK7 by the SUMO3/4 protease SENP3 activates the LPS-induced TLR4 signaling pathway (25). SUMOylation of NR4A1 controls inflammatory cytokine signaling and macrophage cell death (43). Moreover, MafB SUMOylation not only enhances MafB-driven macrophage differentiation but also inhibits cell cycle progression of myeloid progenitor cells (25). In our study, RNA sequencing (RNA-Seq) of intratumoral TAMs along with SUMOylation assays identified STAT4 as a SUMOylation target involved in the regulation of macrophage antitumor activity. STAT4 can be ubiquitinated by the ubiquitin E3 ligase SLIM and degraded by the ubiquitin-proteasome system. The relationship between SUMOylation and ubiquitination is complicated, and lysine residues are the binding sites for both types of posttranslational modification**?)**. Therefore, ubiquitin and SUMO may concurrently bind to the same lysine residue. Generally, ubiquitination leads to substrate degradation while SUMOylation prevents ubiquitin binding and enhances substrate stability. However, in certain cases, SUMOylation also targets a protein for degradation via the ubiquitin-proteasome–dependent system (46). Herein we identified lysine 350 of STAT4 as the major SUMOylation site, and more critically, the level of STAT4 ubiquitination decreased and the protein stability of STAT4 increased once this site was mutated, indicating that SUMOylation promotes STAT4 degradation. Nevertheless, additional studies are necessary to further illuminate the interaction between ubiquitination and SUMOylation of STAT4, since the STAT4 ubiquitination sites have not been determined to date.

STAT4 is required for IL-12 and IL-18 to induce the production of inflammatory cytokines such as IFN-γ and TNF-α (29, 47). There is also a positive-feedback loop in which IL-12, via STAT4, increases the expression of its receptor IL-12R and the receptor for IL-18. Therefore, STAT4 acts as a key proinflammatory transcription factor in macrophages, which then induce the activation of adaptive immune cells (47). In this report, we demonstrate evidence that *Ubc9* deficiency favors macrophage M1 program, which renders TAMs more potent to stimulate CD8^+^ T cell proliferation and activation. We further proved that CD8^+^ T cells are mainly responsible for the suppression of PCa progression. Together, these findings support that deciphering the interaction between TAMs and CD8^+^ T cells is of great importance to understand the pathogenesis of PCa progression.

Indeed, PCa is defined as a “cold” tumor because of insufficient intratumoral CD8^+^ T cell infiltration/activation coupled with the lack of success of anti–PD-1 and/or anti–CTLA-4 therapy. To exhibit cancer cell cytotoxicity, CD8^+^ T cells must first accumulate in the prostate tumor, maintain physical contact with tumor cells, and secrete antitumor cytokines. CD8^+^ T cells also need to respond adequately to tumor antigens by receiving activation signals from antigen-presenting cells, such as TAMs and dendritic cells (48). We found that the enhanced activation and antigen presentation induced by a UBC9 inhibitor, 2-D08, sensitized TAMs to promote CD8^+^ T cell–mediated cytotoxicity, and 2-D08 could also directly act on PCa cells to inhibit their proliferation. Interestingly, we further found that CD8^+^ T cells activated by *Ubc9^–/–^* TAMs manifested enhanced PD-1 expression. These discoveries prompted us to assume that inhibition of UBC9 may also synergistically enhance the therapeutic effect of anti–PD-1 antibodies on PCa progression. Indeed, 2-D08 combined with anti–PD-1 therapy remarkably suppressed prostate tumor progression along with a more pronounced increase of CD8^+^ T cell frequency and a higher percentage of IFN-γ^+^ CD8^+^ T cells in the prostate tumors.

In summary, macrophages deficient in *Ubc9* display enhanced STAT4 expression and transcriptional activity, which favors TAM M1 program and CD8^+^ effector T cell activation. Given that inhibition of the SUMOylation process in tumors would generate a global effect on other TME components, we cannot exclude the possibility that additional mechanisms other than TAMs and tumor cells may also exist with regard to the chemical blockade of UBC9. Nonetheless, in this report, our focus is to address the effect of UBC9 on macrophage polarization relevant to the activation of CD8^+^ effector T cells. In this case, we provided molecular and cellular insights into the manipulation of TAM protumoral activities and suggested a valuable approach to reshape the antitumor TME. Overall, our data support that the use of UBC9 inhibitor along with PD-1 blockade could be a viable approach against PCa in clinical settings.

## Methods

### Mouse model.

*Ubc9^fl/fl^* mice were generated as described previously and were backcrossed with *LyzM-Cre* transgenic mice to generate mice with selective deletion of *Ubc9* in macrophages (*LyzM-Cre^+^Ubc9^fl/fl^*) (21). Eight- to twelve-week-old male littermates (*LyzM-Cre^−^Ubc9^fl/fl^* mice) were used as controls. Wild-type (WT), CD45.2^+^ congenic C57BL/6, OT-I mice were purchased from The Jackson Laboratory (Shanghai, China). Male nude mice (8 weeks old) were purchased from Beijing Huafukang Bioscience. All mice were housed in the Tongji Hospital Animal Center with a 12-hour light/12-hour dark cycle in a specific pathogen–free facility.

### Bioinformatics analysis.

Bioinformatics analyses were performed using the RStudio 4.1.0 mRNA expression profile (fragments per kilobase per million mapped reads [FPKM] value matrix, *n* = 489) of prostate adenocarcinoma obtained from the Xena portal, and the FPKM matrix was transformed into a transcripts per million (TPM) value matrix and log_2_-transformed for subsequent analysis. Samples were divided into *UBC9^hi^* and *UBC9^lo^* groups based on the median expression value of *UBC9*. DEG analysis was performed between *UBC9^hi^* and *UBC9^lo^* groups using the limma package, and DEGs belonging to the “antigen presentation (MHC I related),” “immune checkpoint,” “innate immunity,” “T cell activation,” and “tumor progression” gene sets with adjusted *P* value less than 0.05 were selected out and *z*-score-transformed before being visualized in the heatmap. Gene set enrichment analysis (GSEA) was performed using the Gene Set Variation Analysis (GSVA) package (http://vip.sangerbox.com/login.html). CIBERSORT was performed to uncover the expression matrix to estimate the infiltration proportion of 22 immune cell types (https://cibersortx.stanford.edu). Samples with P value less than 0.05 were excluded because the estimation results did not meet the stability standard, and then 93 samples were used for comparison.

### Cell culture.

The RM-1 mouse PCa, B16F10, and B16-OVA transgenic melanoma cell lines (B16F10 melanoma cells stably expressing chicken ovalbumin) were cultured in complete DMEM (Sigma-Aldrich; DMEM supplemented with 10% FBS and penicillin/streptomycin). RM-1 and B16F10 cell lines were obtained from ATCC, and B16-OVA was provided by Cheng-Tao Jiang (South China University of Technology, Guangzhou, China). Bone marrow–derived macrophages (BMDMs) were differentiated with 20 ng/mL M-CSF in vitro for 7 days. The differentiated BMDMs were treated with 50 ng/mL LPS (PeproTech, Wuhan, China) or untreated for the indicated times. The cells were then harvested for quantitative real-time PCR, flow cytometry, and Western blot analysis. BMDMs were cultured in RPMI 1640 (Gibco, Shanghai, China) supplemented with 10% FBS (Gibco) and 1% antibiotics (penicillin/streptomycin) (Beyotime). The GFP-labeled adenoviruses (Vector, *Stat4*-WT, *Stat4*-K350R, *Ubc9*-overexpressing) were packaged by Dianjun Biotech Co. Ltd. BMDMs were transduced with the empty control virus (Vector) or the adenovirus carrying the FLAG-tagged murine *Stat4* gene (*Stat4*-WT) or mutant gene (*Stat4*-K350R) and treated with IL-12 (10 ng/mL) for the indicated periods of time before further analysis.

### Antibodies and reagents.

Recombinant murine LPS (L4130) and recombinant murine M-CSF (catalog 315-02-250) were from PeproTech (Wuhan, China). Ovalbumin (OVA 257–264; catalog S7951) was from Sigma-Aldrich. Anti-STAT4 (2653S), anti-FLAG (catalog 2368), and anti-ubiquitin (catalog 3936s) were from Cell Signaling Technology. Anti-CD8 (catalog ab217344) and anti-UBC9 (catalog ab75854) were from Abcam. Anti-CD68 (catalog 66231-2-Ig) was from Proteintech (Wuhan, China). Anti-SUMO1 was from Youke Group (Shanghai, China). BV510–anti–mouse CD45.2 (catalog 109837), FITC–anti–mouse F4/80 (catalog 123108), PE–anti–mouse CD11b (catalog 101208), BV421–anti–mouse F4/80 (catalog 123132), PE/Cy7–anti–mouse CD86 (catalog 105014), APC–anti–mouse MHC I (catalog 116418), FITC–anti–mouse MHC II (catalog 107606), FITC–anti–mouse CD4 (catalog 100406), PE/Cy7–anti–mouse CD8 (catalog 140416), APC–anti–mouse PD-1 (catalog 135210), APC–anti–mouse IFN-γ (catalog 505810), BV421–anti–mouse TNF-α (catalog 506327), APC–anti–mouse granzyme B (catalog 372204), and PE–anti–mouse Ki67 (catalog 151210) were from BioLegend. Dynabeads Protein G (catalog 1004d) was purchased from Invitrogen. *N*-Ethylmaleimide (catalog 23030.0) was purchased from Sigma-Aldrich. The GFP-labeled *Stat4*-WT (FLAG tagged), *Stat4*-K350R (FLAG tagged), and *Ubc9*-overexpressing adenoviruses were from Dianjun Biotech Co. Ltd. Percoll (catalog 65455-52-9) was purchased from Solarbio Life Science Co. CCK-8 (catalog BS350B) was purchased from Biosharp Life Science Co. InVivoMAb anti–mouse PD-1 (catalog BE0146) and anti–mouse CD8 (catalog BE0004) were purchased from Bio X Cell Co. The UBC9 inhibitor 2-D08 (catalog HY-114166), which inhibits protein SUMOylation by preventing the transfer of SUMO from the UBC9-SUMO thioester to the substrates, was purchased from MCE Biotechnology (Shanghai, China). The SUMOylation-activating enzyme E1 inhibitor TAK-981 (catalog HY-111789) and the proteasome inhibitor MG-132 (catalog HY-13259) were purchased from MCE Biotechnology, while cycloheximide (CHX) was from MedChemExpress (catalog HY-12320).

### Xenograft tumor model.

To check the effect of the SUMOylation inhibitor TAK-981, C57BL/6 mice were inoculated s.c. with 1 × 10^6^ RM-1 cells into the right rear flank. All mice were divided randomly into 2 groups, and tumor sizes were measured every 3 days. The intervention group was treated with TAK-981 (7.5 mg/kg, every 3 days, intratumoral injection), while the remaining group was injected with DMSO as a control for 2 weeks.

For experiments with transgenic mice, male C57BL/6 WT or *Ubc9^–/–^* mice were injected s.c. with 1 × 10^6^ RM-1 cells. Tumor growth was measured every 3 days after tumor inoculation using digital vernier calipers. Mice were sacrificed 14 days after inoculation, and tumors were excised and processed for other experiments. Additionally, for CD8^+^ T cell depletion, an anti-CD8 antibody (200 μg per mouse) was administered (i.p.) starting 3 days before tumor implantation and repeated every 3 days until mouse sacrifice.

For experiments with 2-D08, each mouse was injected s.c. with 1 × 10^6^ RM-1 cells into the right rear flank. All mice were divided randomly into 2 groups, and tumor sizes were measured every 3 days. The intervention group was treated with 2-D08 (10 mg/kg, every 3 days, intratumoral injection), while the remaining group was injected with DMSO as a control for 2 weeks (in either C57BL/6 or nude mice). Furthermore, an anti–PD-1 antibody (250 μg per mouse) was injected i.p. every 3 days, starting from day 3 after tumor implantation in mice receiving combination therapy of 2-D08 plus anti–PD-1.

For experiments with macrophage adoptive transfer, B16-OVA cells were grown in DMEM containing 10% FBS. A total of 2 × 10^5^ B16-OVA cells in 100 μL PBS were injected s.c. into the right rear flank of 8-week-old male C57BL/6 mice (*n* = 7 mice per group). WT and *Ubc9^–/–^* macrophages (5 × 10^5^ cells) were pulsed with LPS and OVA (10 μg/mL) overnight before para-tumor injection on days 3 and 10. In another set of experiments, mice were injected s.c. with 1 × 10^6^ RM-1 cells to establish the PCa model. WT BMDMs were pretreated with *Stat4* siRNA targeting the 3′-untranslated region (3′-UTR) to knock down the endogenous *Stat4*, and then transduced with FLAG-tagged *Stat4-*WT or *Stat4*-K350R adenoviruses. The *Stat4*-WT– and *Stat4*-K350R–transduced macrophages (5 × 10^5^ cells) were treated with LPS overnight before para-tumor injection on days 3 and 10 as above.

The tumor volume was recorded every 3 days using the digital vernier calipers and calculated according to the formula *V* = (length × width^2^)/2. Tumor weights were determined with electronic scales. After the mice were sacrificed, solid tumors were retrieved and processed using a tumor dissociation kit (MACS, Miltenyi Biotec, USA) for further analysis.

### Isolation of tumor-infiltrated leukocytes.

On day 14 after tumor inoculation, tumors were harvested, minced, dissociated with a tumor dissociation kit (MACS), and filtered through a 40 μm strainer to obtain single-cell suspensions. Red blood cells were lysed with RBC lysis buffer (Sigma-Aldrich). Tumor-infiltrated lymphocytes were isolated by Percoll gradient following the manufacturer’s protocol (Solarbio).

### Flow cytometry analysis.

Cells were incubated with fluorescently labeled antibodies on ice in the dark for 30 minutes and then washed with FACS buffer (2% BSA in PBS). For intracellular staining, cells were permeabilized and stained with corresponding antibodies. Cells were analyzed using an LSRFortessa (BD Biosciences) equipped with Diva. Data were further analyzed using FlowJo software.

### RNA extraction and quantitative real-time PCR analysis.

Total RNA was isolated from tumor tissues and BMDMs using Trizol reagent (Takara). For mRNA analysis, an aliquot containing 1 μg of total RNA was reverse-transcribed using a cDNA synthesis kit (Takara). Real-time qPCR was performed using SYBR Green PCR master mix (Applied Biosystems) in an ABI Prism 6000 Sequence Detection System (Applied Biosystems). The following primers were used: major histocompatibility complex I (*Mhc-i*): forward 5′-AAGTGTCTGATGTTCCCTGTG-3′, reverse 5′-ATGTCCCTCAGTGTTTGGC-3′; cluster of differentiation 86 (*Cd86*): forward 5′-CCTCAAGTTTCCATGTCCAAGGC-3′, reverse 5′-GAGGAGAGTTGTAACGGCAAGG-3′; tumor necrosis factor-α (*Tnf-a*): forward 5′-ACTGAACTTCGGGGTGATCG-3′, reverse 5′-GGCTACAGGCTTGTCACTCG-3′; interferon-γ (*Ifn-g*): forward 5′-TGGCTCTGCAGGATTTTCAT-3′, reverse 5′-TCAAGTGGCATAGATGTGGA-3′; and β-actin: forward 5′-AGCCATGTACGTAGCCATCC-3′, reverse 5′-CTCCAGCTGTGGTGGTGAA-3′. The relative expression level of each gene was normalized by β-actin and calculated with the 2^−ΔΔCt^ method as previously reported (49).

### Western blot analysis.

Cell lysates were prepared using RIPA buffer (Servicebio) containing a protease inhibitor cocktail (Roche). Western blot analysis of target proteins was conducted as described previously using the corresponding primary antibodies, followed by exposure to an HRP-conjugated secondary antibody. The reactive bands were visualized using ECL Plus reagents (Servicebio), and the relative intensity of reactive bands was analyzed using ImageJ software (NIH).

### Confocal microscopy analysis.

Immunofluorescence was conducted to identify the expression of UBC9 and CD68 in human PCa tumor tissues, and the subcellular localization of FLAG-STAT4 and GFP in BMDMs. BMDMs were cultured as described above, and nuclei were stained by DAPI. UBC9, CD68, FLAG-STAT4, and GFP were labeled by primary antibody, followed by fluorescently labeled secondary corresponding antibody IgG (H+L) (1:300; Jackson ImmunoResearch Laboratories). The stained tissue and BMDMs were imaged and analyzed under a confocal microscope (FV1000, Olympus) at ×60 magnification.

### Cell viability analysis.

RM-1 cells were plated in a 96-well plate with a density of 3 × 10^3^ cells per well, and 2-D08 or TAK-981 was added into RM-1 cultures with indicated concentrations. After 48 hours of treatment, CCK-8 reagent was incubated for 2 hours, and the absorbance was measured at 450 nm with a microplate reader.

### ELISA assay.

The levels of IFN-γ and TNF-α in the supernatant of macrophages were determined using an ELISA kit purchased from eBioscience (Shanghai, China).

### Stat4 siRNAs.

*Stat4* siRNAs targeting sequences located within the 3′-UTR were synthesized by Dianjun Biotech Co. Ltd. Sequences of siRNA were as follows: siRNA-3′-UTR-#1: forward 5′-CUUUACCAUAGAUCACAAUUUdTdT-3′, reverse 5′-AAAUUGUGAUCUAUGGUAAAGdTdT-3′; siRNA-3′-UTR-#2: forward 5′-CGGCUUUGUAAAUACCAGUUUdTdT-3′, reverse 5′-AAACUGGUAUUUACAAAGCCGdTdT-3′; siRNA-3′-UTR-#3: forward 5′-AGAUGAAACUGGAGAGUGUdTdT-3′, reverse 5′-ACACUCUCCAGUUUCAUCUdTdT-3′. The mixed siRNA sequences were applied to knock down endogenous *Stat4*.

### In vitro SUMOylation assay.

BMDMs were first transduced with either FLAG-negative control (FLAG-NC) or *Stat4*-WT and *Stat4*-K350R adenoviruses, and stimulated by IL-12 (10 ng/mL) overnight. After washes with ice-cold PBS, the cells were lysed on ice for 30 minutes in IP lysis buffer (50 mM Tris-HCl, pH 7.5, 150 mM NaCl, 1% NP-40, 5 mM EDTA, 0.1% SDS) containing protease inhibitors (10 μg/mL aprotinin, 10 μg/mL leupeptin, and 1 mM PMSF), phosphatase inhibitors (5 mM sodium pyrophosphate and 1 mM Na_3_CO_4_), and 20 mM *N*-ethylmaleimide. The cell lysates were precleared with protein G agarose beads for 1 hour, then incubated with 5 μg anti-FLAG antibody overnight, and proteins were immunoprecipitated for an additional 4 hours at 4°C with protein G beads. The resulting products were then probed with a SUMO1 polyclonal antibody for immunoblotting analysis.

### STAT4 stability analysis.

RAW264.7 cells were first transfected with a siRNA to knock down the endogenous *Stat4*, followed by transduction of adenoviruses carrying FLAG-tagged *Stat4*-WT or *Stat4*-K350R. The transduced cells were next stimulated by IL-12 (10 ng/mL) overnight, followed by MG-132 treatment (20 μM) for 6 hours. Cell lysates were prepared after 48 hours of transduction and subjected to coimmunoprecipitation using a FLAG antibody, and the resulting products were used for Western blotting using a ubiquitin antibody. For the cycloheximide (CHX, MedChemExpress) chase experiment, CHX was added into the cultures to prevent novel protein synthesis at indicated time points. The cell lysates were then used for Western blotting to compare the differences of *Stat4*-WT and *Stat4*-K350R degradation using the established techniques (22).

### CD8^+^ T cell coculture assays.

For antigen-specific macrophage–CD8^+^ T cell coculture, WT or *Ubc9^–/–^* BMDMs were prepared and pulsed with LPS and OVA 257–264 for 4 hours as described above. Naive CD8^+^ T cells were isolated from OT-I mice and cocultured with pulsed macrophages (1:5 macrophage/T cell ratio) for another 3–5 days to check the proliferation and activation of T cells by flow cytometry. For antigen-nonspecific macrophage–CD8^+^ T cell coculture, BMDMs were pretreated with *Stat4* siRNA targeting the 3′-UTR to knock down the endogenous *Stat4*, and then transduced with *Stat4*-WT or *Stat4*-K350R adenoviruses. The *Stat4*-WT– and *Stat4*-K350R–transduced macrophages were stimulated with LPS overnight, and then cocultured with naive CD8^+^ T cells (1:5 macrophage/T cell ratio) in the presence of 1 μg/mL anti-CD3 antibody for another 3–5 days. For tumor cell–CD8^+^ T cell coculture, naive CD8^+^ T cells were isolated from OT-I mice and cocultured with 2-D08– or vehicle-pretreated B16-OVA cells (1:5 B16-OVA/T cell ratio) for 3–5 days to check the proliferation and activation of T cells as above.

### Histological analysis.

Human PCa tissues and mouse tumor tissues were harvested and fixed in 4% paraformaldehyde and then were embedded in paraffin. Immunofluorescence and immunohistochemistry were performed as previously described (50).

### Data availability.

The raw RNA-Seq data were deposited in the NCBI’s Sequence Read Archive public repository (SRA PRJNA916279).

### Statistics.

All in vitro experiments were conducted with at least 3 independent replications. Comparisons between groups were performed using the unpaired Student’s *t* test (2 tailed) and 1-way ANOVA or 2-way ANOVA (Šidák’s comparison test with selected pairs). Survival was determined by Kaplan-Meier method, and survival curves between different groups were calculated by log-rank test. Statistical analysis of the data was conducted using GraphPad Prism 5 software (GraphPad Software Inc.). A *P* value of less than 0.05 was considered statistically significant.

### Study approval.

All animal care and experimental procedures were approved by the Animal Care and Use Committee of Tongji Hospital, Tongji Medical College, Huazhong University of Science and Technology (TJH-201901019), and conducted in accordance with NIH guidelines. The studies in human samples were approved by the Ethics Committee of Tongji Hospital (2019CR101).

## Author contributions

JX, FS, FXW, HFZ, BL, SGW, and ZHW conceived the project. JX, YNW, PZ, HFZ, YG, TTY, JHL, CLY, SJR, ZZX, SM, and QZ contributed to interpretation of data. JX, FS, FXW, YX, CGY, YL, BL, SGW, and ZHW performed experiments. PZ, YX, FS, and YL performed data and statistical analysis. CYW, SGW, and ZHW supervised the project. JX, FS, and YNW wrote the original draft of the manuscript. SGW, ZHW, and CYW wrote, reviewed, and edited the manuscript.

## Supplementary Material

Supplemental data

## Version 1 01/10/2023

In-Press Preview

## Version 2 02/15/2023

Electronic publication
