# Supplementary material for: UBC9 deficiency enhances immunostimulatory macrophage activation and subsequent antitumor T cell response in prostate cancer
Source: J Clin Invest. 2023 Feb 15;133(4):e158352. doi: 10.1172/JCI158352 (PMC9927932; doi:10.1172/JCI158352)
Supplement: Supplemental data [file jci-133-158352-s143.pdf]

Supplementary Figures:

Supplemental Figure 1.

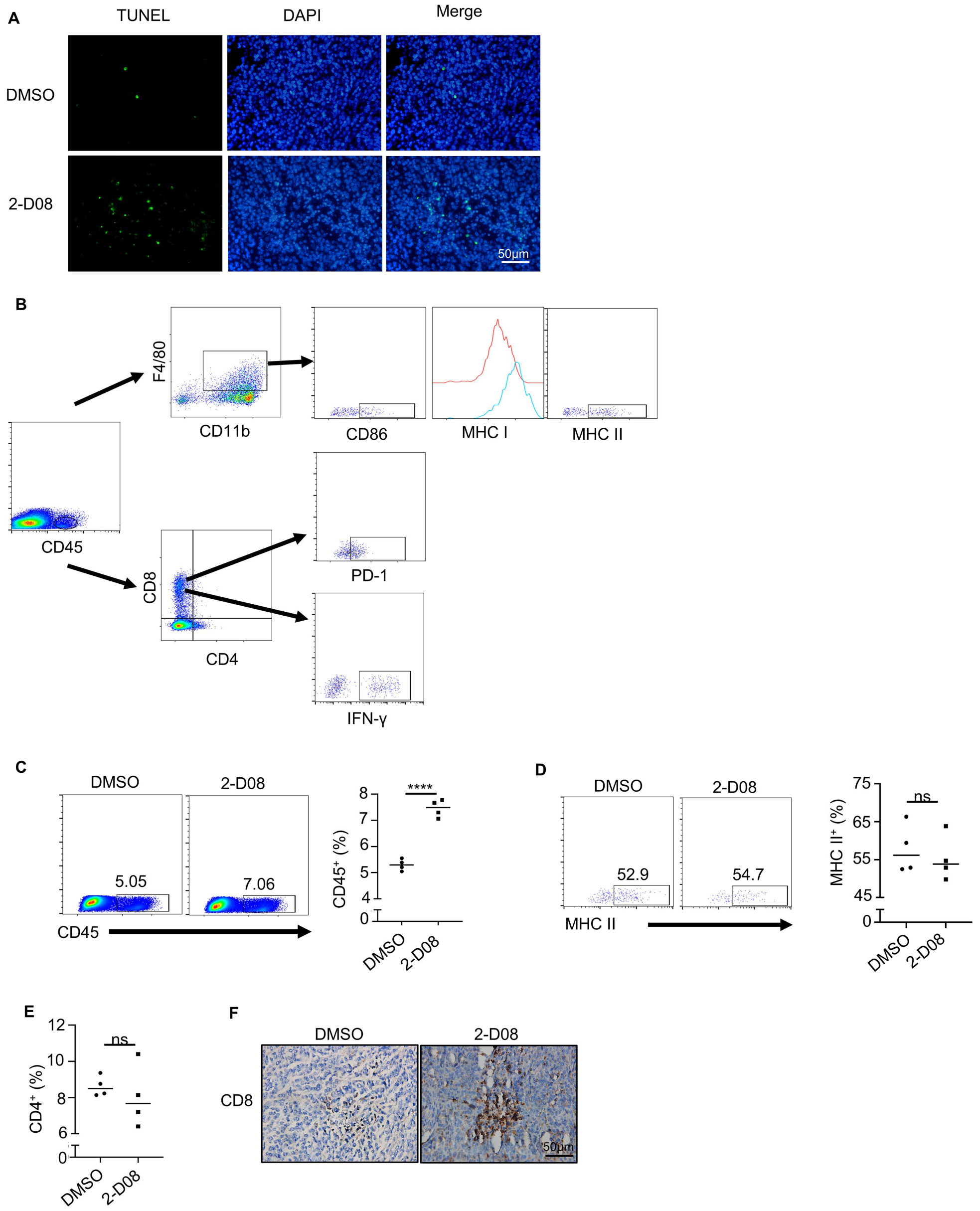

2-D08 promotes prostate tumor apoptosis and facilitates immune cell infiltration. (**A**) TUNEL staining of tumors in the DMSO and 2-D08-treated mice. (**B**) Gating strategy for infiltrating immune cells in prostate tumors. (**C**, **D** and **F**) $CD45^+$ immune cells (**C**), MHC II^+ macrophages (**D**), and $CD4^+$ T cells (**E**) in tumors compared in the DMSO and 2-D08 treated mice, respectively. (**F**) IHC staining of $CD8^+$ T cells in the two groups. (**A**) and (**F**) represent results from three independent experiments. Data in (**C-E**) represent mean ± SEM and were analyzed by Student's *t*-test (n = 4 / group). ****P < 0.0001; ns, not significant.

Supplemental Figure 2.

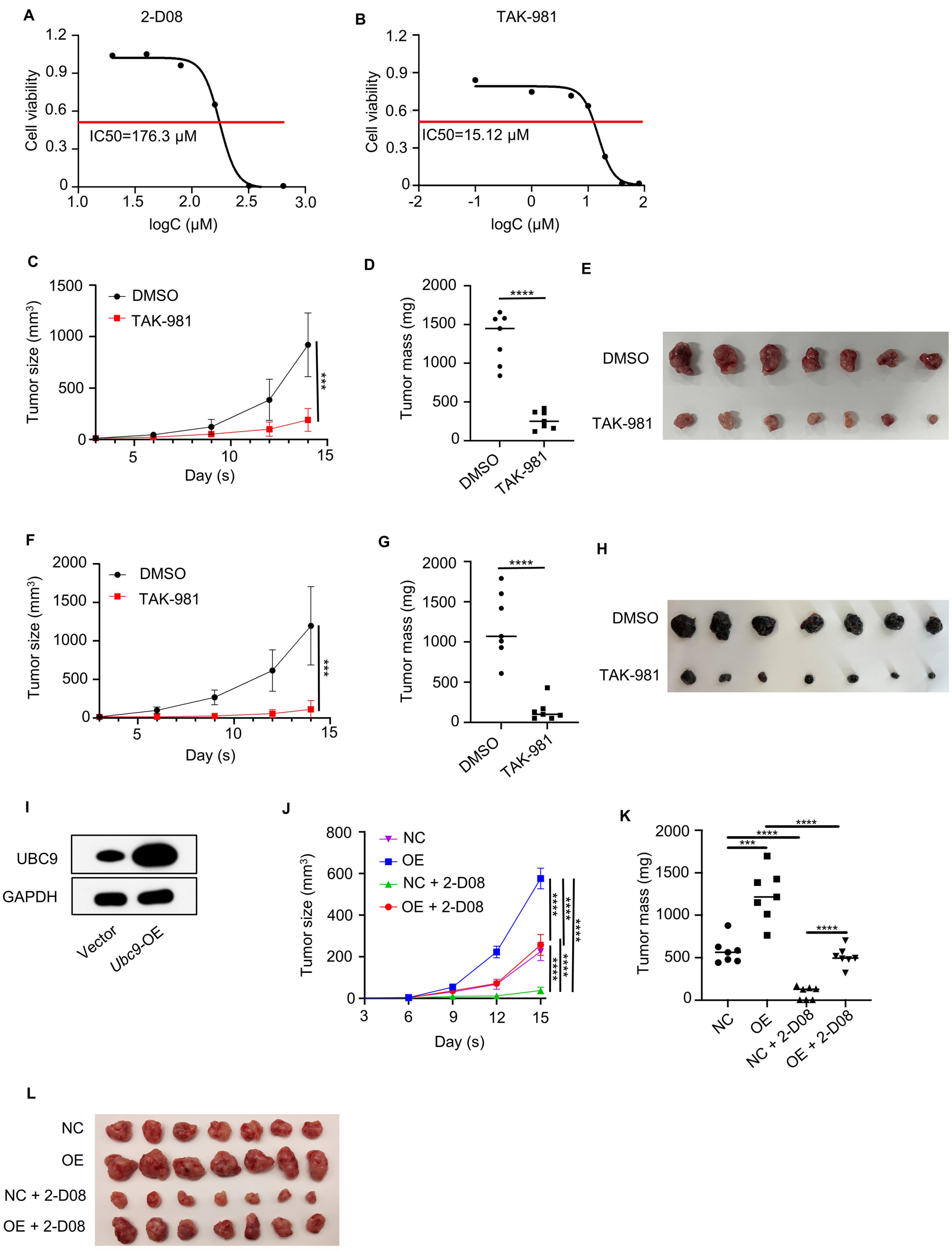

SUMOylation inhibitors repress tumor cell proliferation and progression. (**A** and **B**) The cell viability of prostate cancer cell line RM-1 treated with SUMOylation inhibitors, 2-D08 (**A**) and TAK-981 (**B**) *in vitro*. (**C**, **D** and **E**) Prostate tumor growth curve (**C**) and tumor mass (**D** and **E**) in nude mice at day 14 treated with DMSO and TAK-981 *in vivo*. (**F**, **G** and **H**) Melanoma tumor growth curve (**F**) and tumor mass (**G** and **H**) in nude mice at day 14 treated with DMSO and TAK-981 *in vivo*. (**I**) Western

blotting assay on the RM-1 cells transduced with *Ubc9* adenovirus (*Ubc9*-OE) against vector (vector as control). (**J**, **K** and **L**) Prostate tumor growth curve (**J**) and tumor mass (**K** and **L**) of RM-1 transduced with *Ubc9*-OE or control (NC), treated with 2-D08 in nude mice at day 14 *in vivo,* $n = 7$ per group. Data represent mean ± SEM and were analyzed by Student's *t*-test. ***$P < 0.001$; ****$P < 0.0001$; ns, not significant.

Supplemental Figure 3.

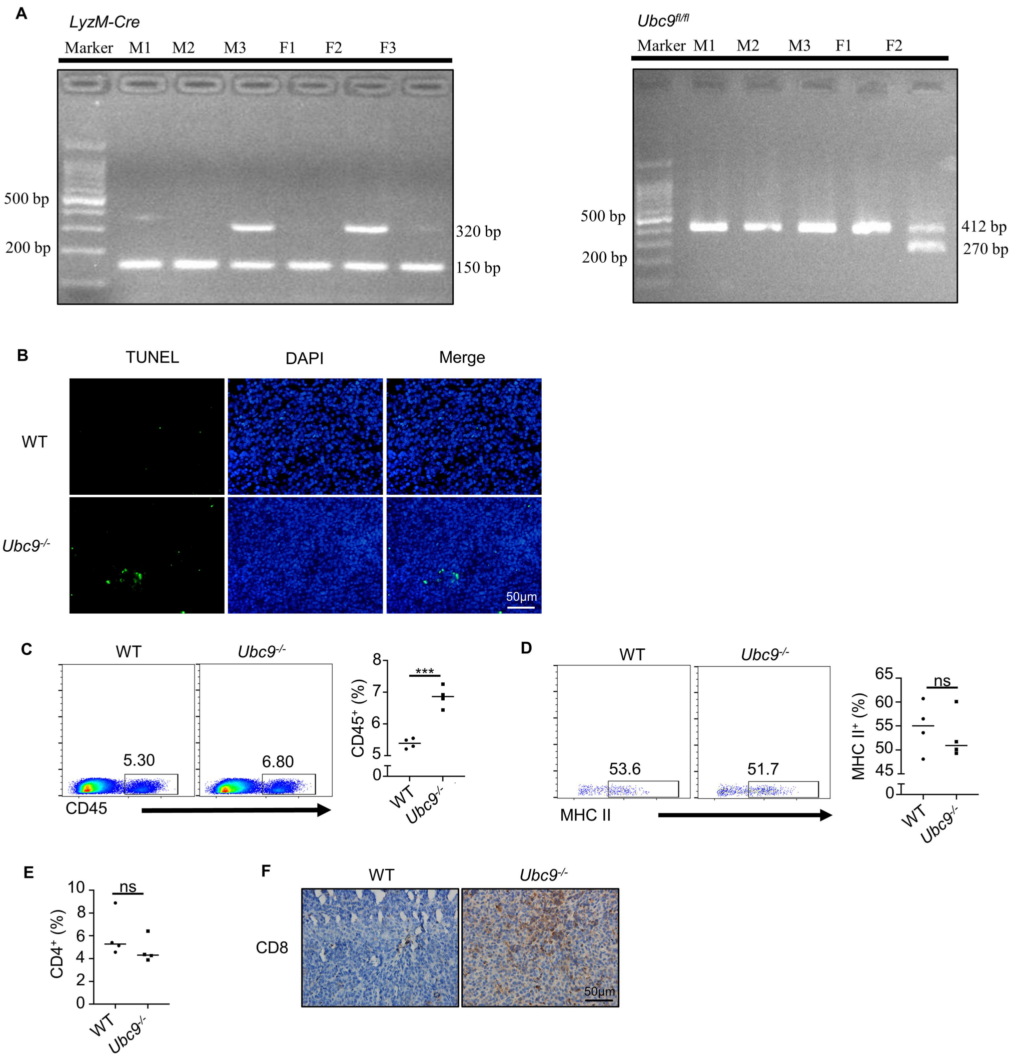

***Ubc9* deficiency in macrophages accelerates prostate tumor apoptosis and immune cell infiltration.** (**A**) Genotyping for the *Lyzm-Cre* and *Ubc9$^{fl/fl}$*. (**B**) TUNEL staining of tumors from WT and *Ubc9$^{-/-}$* mice. (**B, C** and **D**) CD45^{+} immune cells (**C**), MHC II^{+} macrophages (**D**), and CD4^{+} T cells (**E**) in tumors from WT and *Ubc9$^{-/-}$* mice, respectively. (**F**) IHC staining of CD8^{+} T cells in the two groups. Representative data from three experiments are shown for (**B**) and (**F**). Data in (**C-E**) represent mean ± SEM and were analyzed by Student's *t*-test (n = 4 / group). ***P < 0.001; ns, not significant.

Supplemental Figure 4.

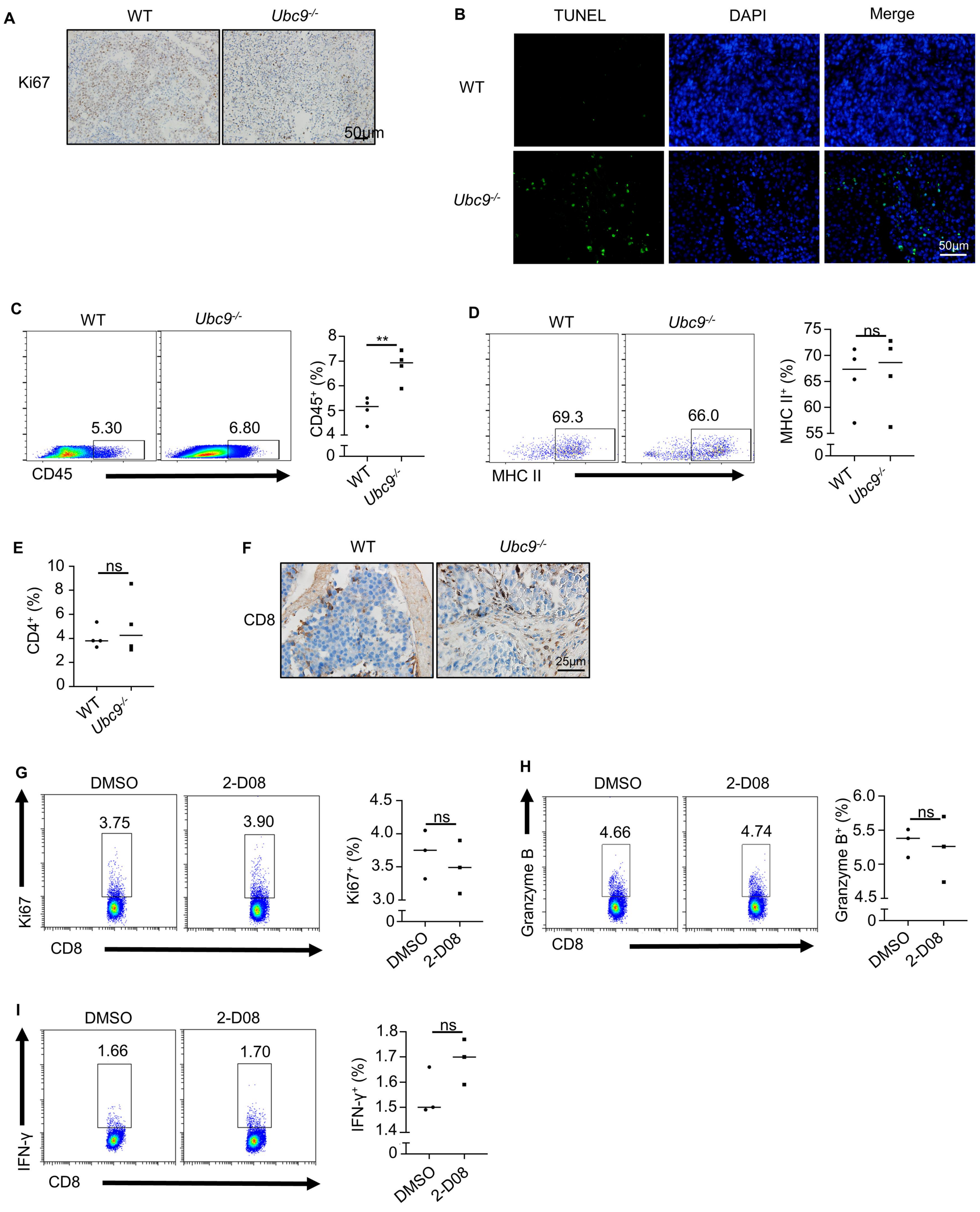

***Ubc9* deficiency in macrophages promotes CD8⁺ T cell infiltration while 2-D08 treated tumor cells fail to enhance CD8⁺ T cell activation. (A and B)** Ki67 staining (**A**) and TUNEL staining (**B**) of B16-OVA tumors adjacently transferred with OVA-pulsed WT or *Ubc9*$^{-/-}$ macrophages, n = 4 per group. (**C**, **D** and **E**) $CD45^+$ immune cells (**C**), MHC II^+ macrophages (**D**), and $CD4^+$ T cells (**E**) in tumors from these two groups, respectively, n = 4 per group. (**F**) IHC staining of $CD8^+$ T cells in the two groups. (**G**, **H** and **I**) The proportions of $Ki67^+$ (**G**), Granzyme B^+ (**H**) and IFN-γ^+ (**I**)

$CD8^+$ T cells in co-culture assay combining B16-OVA pre-treated with DMSO or 2-D08 with OT-I $CD8^+$ T cells, $n = 3$ per group. Representative data from three experiments are shown for (**A**), (**B**) and (**F**). Data in (**C-E**) and (**G-I**) represent mean ± SEM and were analyzed by Student's *t*-test. $^{**}P < 0.01$; ns, not significant.

Supplemental Figure 5.

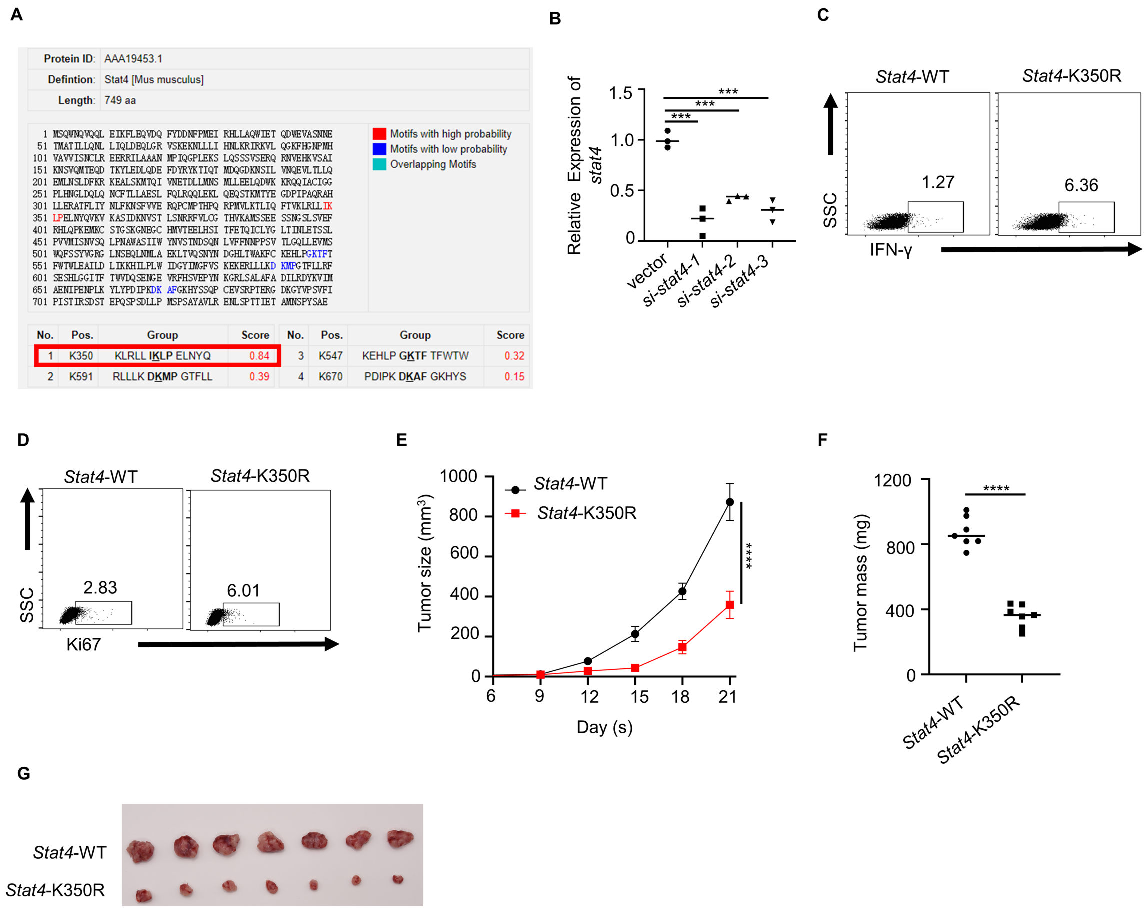

SUMOylation of STAT4 at lysine residue 350 is related to the antitumor function of macrophages. (**A**) SUMOsp2.0 software predicting lysine residue 350 (K350) as a potential site of SUMOylation. (**B**) Endogenous *Stat4* in BMDMs was knocked down by siRNA. (**C** and **D**) Macrophages transduced with *Stat4*-WT or *Stat4*-K350R after endogenous *Stat4* knockdown were co-cultured with CD8^{+} T cells in the presence of anti-CD3. The representative flow cytometry plots for IFN-γ (**C**) and Ki67 (**D**) in CD8^{+} T cells were showed. (**E**, **F** and **G**) *Stat4*-WT or *Stat4*-K350R transduced macrophages were adoptively transferred into the adjacent sites of prostate tumors. RM-1 prostate tumor growth curve (**E**) and tumor mass (**F** and **G**) at day 14 in *Stat4*-WT and *Stat4*-K350R groups, *n* = 7 per group. (**E**) was determined by log-rank test. Data in (**B**) and (**F**) represent mean ± SEM and were analyzed by Student's *t*-test. ***P < 0.001; ****P < 0.0001; ns, not significant.

Supplemental Figure 6.

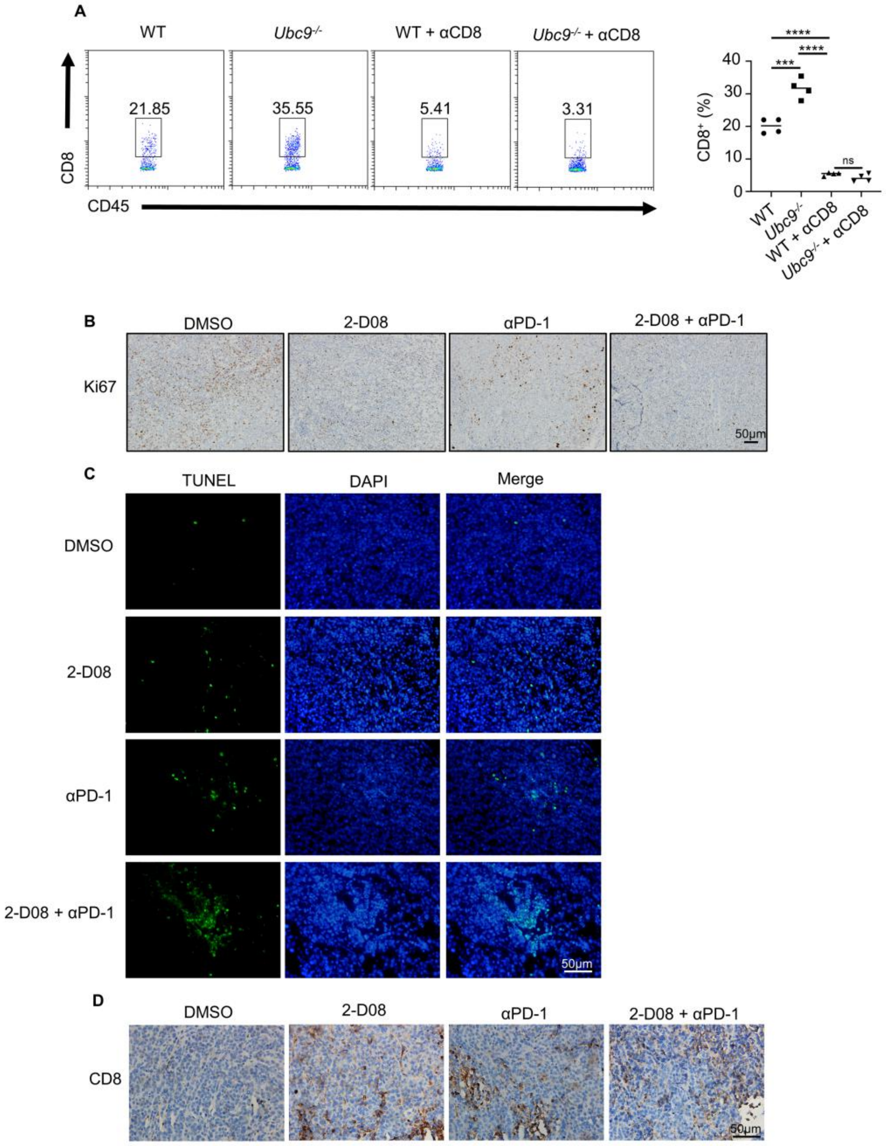

2-D08 exhibited synergistic effect with anti-PD-1 on the inhibition of prostate tumor growth. (**A**) The proportion of $CD8^+$ T cells among $CD45^+$ immune cells following $CD8^+$ T cell depletion in WT and *Ubc9*$^{-/-}$ mice, n = 4 per group. (**B**, **C** and **D**) Ki67 staining (**B**), TUNEL staining (**C**) and IHC staining for $CD8^+$ T cells (**D**) in the tumors from DMSO, 2-D08, anti-PD-1 antibody, or 2-D08 plus anti-PD-1 antibody groups, respectively. Data in (**A**) represent mean ± SEM and were analyzed by Student's *t*-test. ***$P < 0.001$; ****$P < 0.0001$; ns, not significant.

Full unedited gel for Figure 1G

UBC9

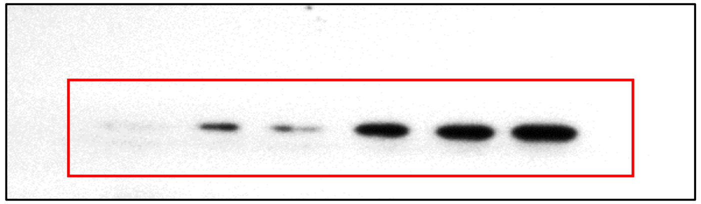

β-actin

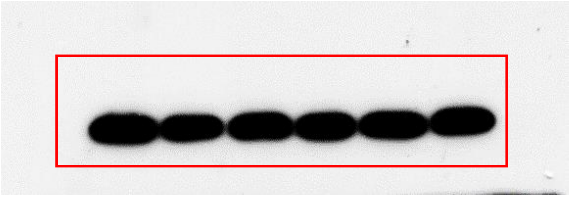

Full unedited gel for Figure 6C

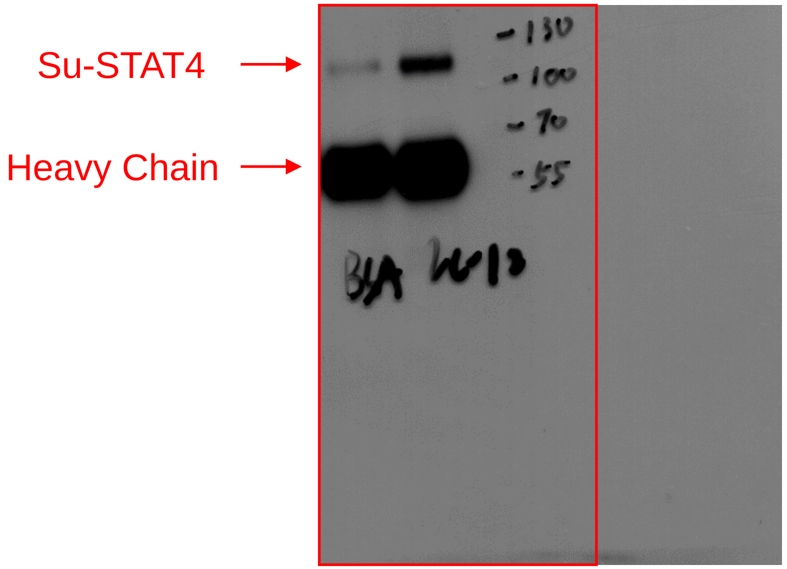

Full unedited gel for Figure 6D

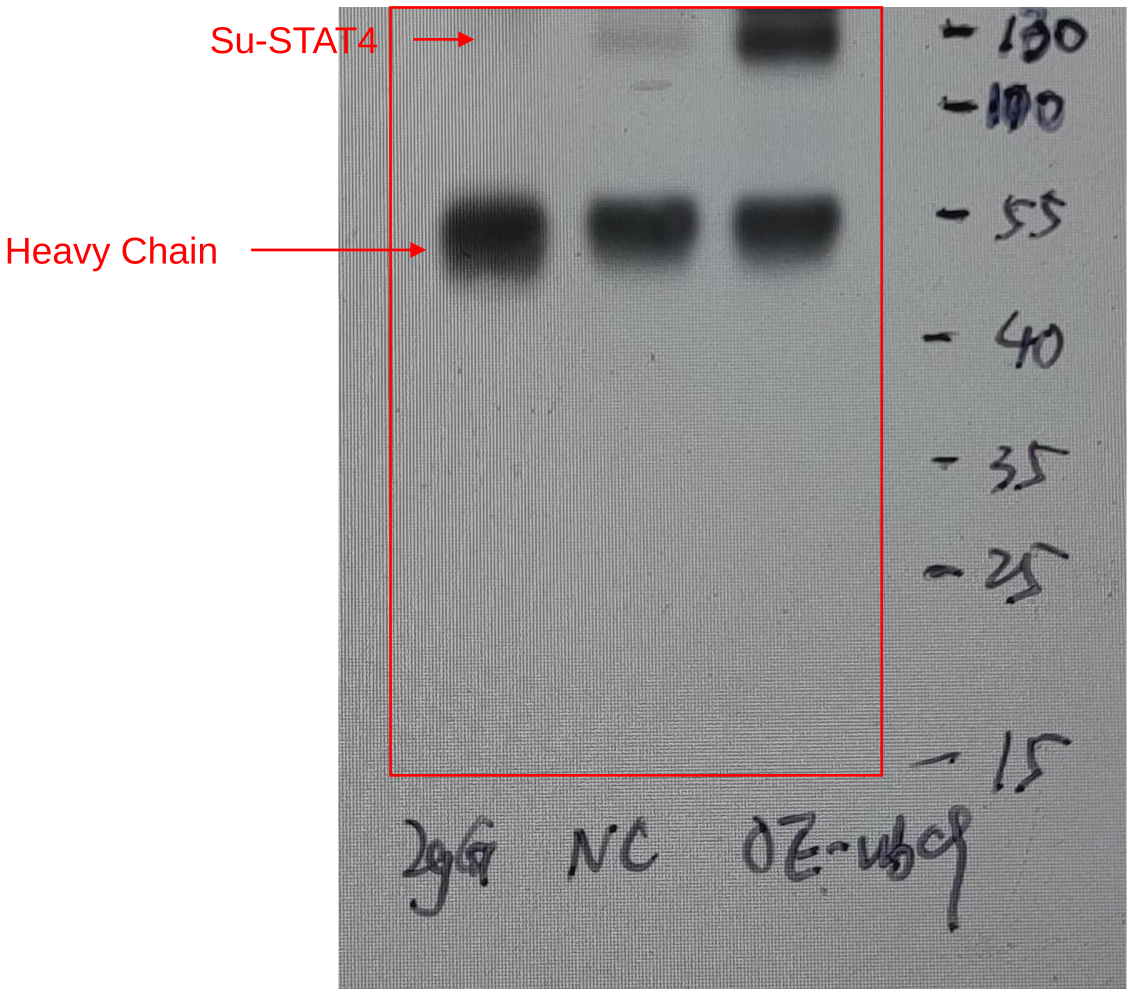

Full unedited gel for Figure 6E

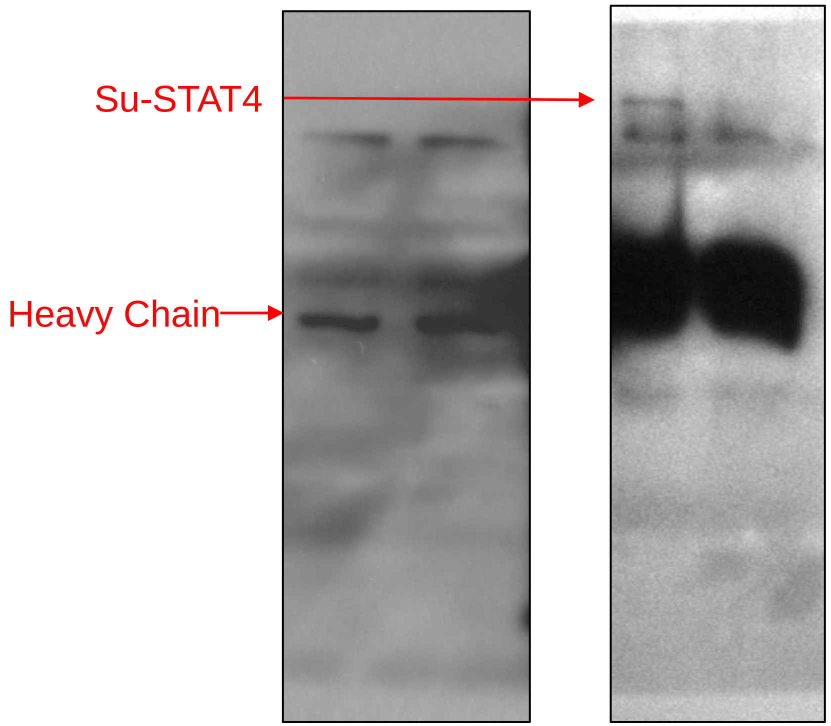

Full unedited gel for Figure 6F

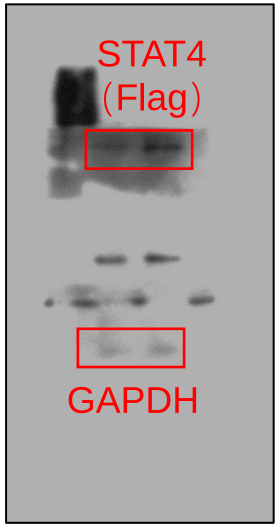

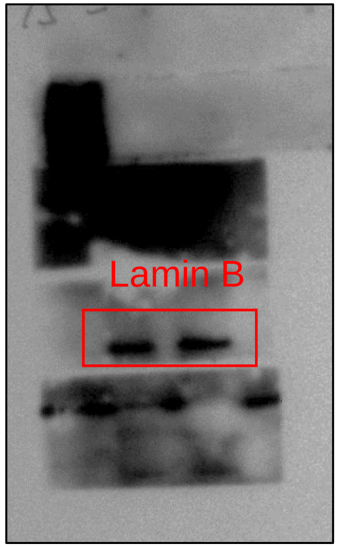

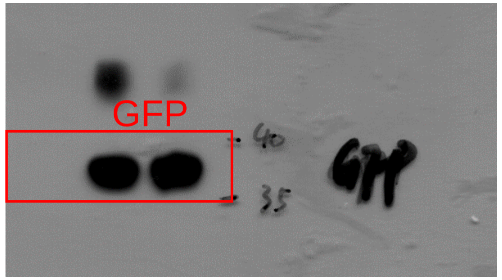

Full unedited gel for Figure 6H

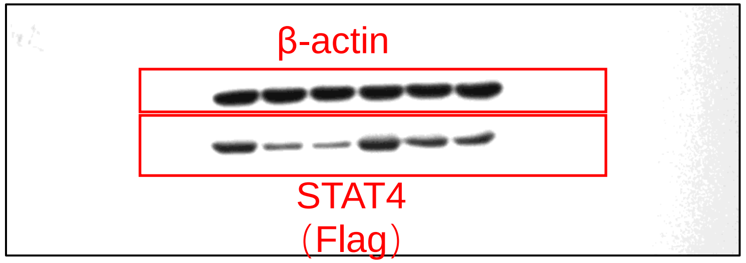

Full unedited gel for Figure 6I

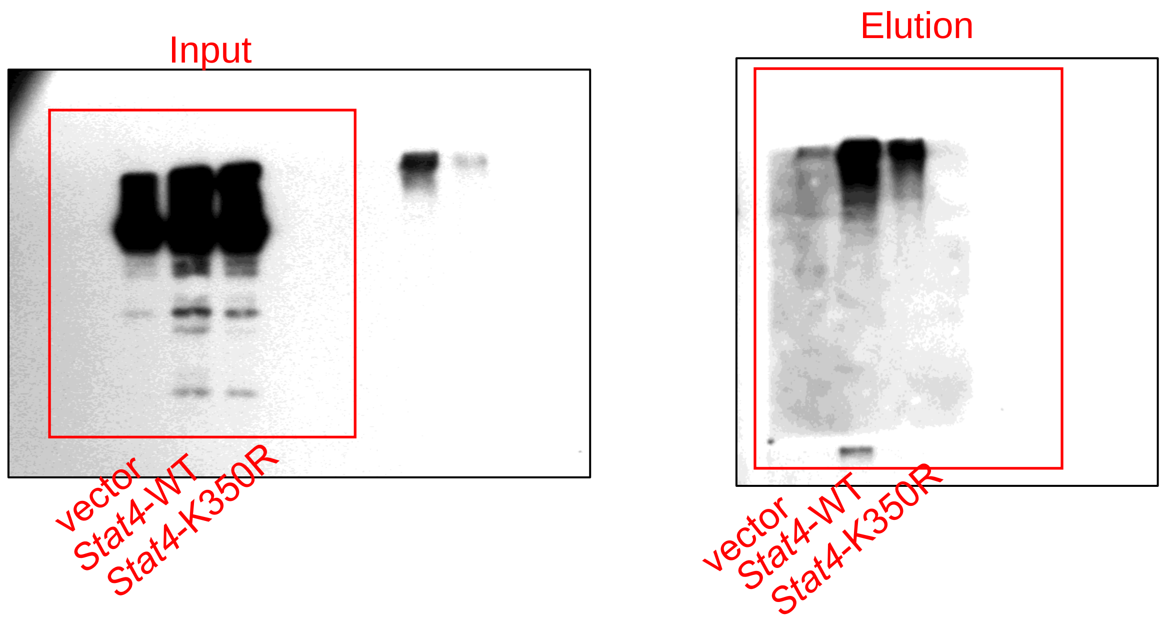

Full unedited gel for Supplementary Figure 2I

UBC9

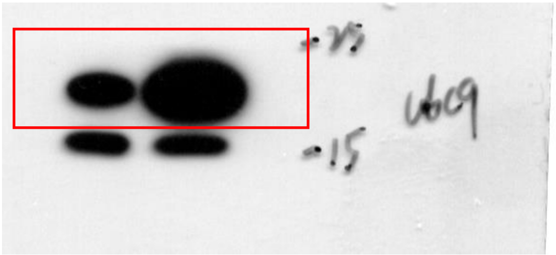

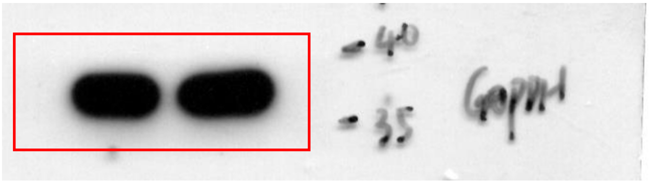

GAPDH
